# Above Room Temperature Ferroelectricity in Epitaxially Strained KTaO_3_

**DOI:** 10.1002/adma.74366

**Published:** 2026-08-11

**Authors:** Tobias Schwaigert, Salva Salmani‐Rezaie, Sankalpa Hazra, Utkarsh Saha, Maya Ramesh, Aiden Ross, Betül Pamuk, Long‐Qing Chen, David A. Muller, Darrell G. Schlom, Venkatraman Gopalan, Kaveh Ahadi

**Affiliations:** ^1^ Platform for the Accelerated Realization Analysis, and Discovery of Interface Materials (PARADIM) Cornell University Ithaca New York USA; ^2^ Department of Materials Science and Engineering Cornell University Ithaca New York USA; ^3^ School of Applied and Engineering Physics Cornell University Ithaca New York USA; ^4^ Kavli Institute at Cornell for Nanoscale Science Cornell University Ithaca New York USA; ^5^ Department of Materials Science and Engineering Ohio State University Columbus Ohio USA; ^6^ Department of Materials Science and Engineering Pennsylvania State University University Park Pennsylvania USA; ^7^ Department of Physics and Astronomy Williams College Williamstown Massachusetts USA; ^8^ Leibniz‐Institut für Kristallzüchtung Berlin Germany; ^9^ Department of Physics Pennsylvania State University University Park Pennsylvania USA; ^10^ Department of Electrical and Computer Engineering Ohio State University Columbus Ohio USA

**Keywords:** condensed matter physics, curie temperature, epitaxy, ferroelectricity, heterojunction, materials science, polarization density, thin film

## Abstract

Epitaxial strain is a powerful means to engineer emergent phenomena in thin films and heterostructures. Here, we demonstrate that ${\mathrm{KTaO}}_{3}$, a cubic perovskite in bulk form, can be epitaxially strained into a highly tunable ferroelectric. ${\mathrm{KTaO}}_{3}$ films grown commensurate to ${\mathrm{SrTiO}}_{3}$ (001) substrates experience an in‐plane strain of −2.1 % that transforms the cubic structure into a tetragonal polar phase with a transition temperature of 475K, consistent with our thermodynamic calculations. We show that the Curie temperature and the spontaneous electric polarization can be systematically controlled with epitaxial strain. Scanning transmission electron microscopy reveals cooperative polar displacements of the potassium columns with respect to the neighboring tantalum columns at room temperature. Optical second‐harmonic generation results are described by a tetragonal polar point group (4mm), indicating the emergence of a global polar ground state. We observe a ferroelectric hysteresis response using metal–insulator–metal capacitor test structures. The results demonstrate a robust intrinsic ferroelectric state in epitaxially strained ${\mathrm{KTaO}}_{3}$ thin films.

## Graphical abstract

Through precise epitaxial engineering, the cubic and paraelectric ${\mathrm{KTaO}}_{3}$ is transformed into a robust ferroelectric. By leveraging lattice‐mismatched substrates to apply controlled epitaxial strain, a stable ferroelectric ground‐state is achieved with a transition temperature as high as 475 K, establishing ${\mathrm{KTaO}}_{3}$ as a highly tunable platform for the next generation of oxide ferroelectric thin films.

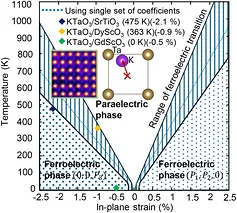

## 1 Introduction

The ability to precisely tune specific properties defines the success of any materials system as a platform for new technologies. The misfit to an underlying substrate can impart an enormous strain to films and heterostructures, exceeding the failure limit of bulk samples and modifying their energy landscape. Consequently, epitaxial strain can be harnessed as a tuning knob for emergent phenomena and functional properties. For example, epitaxial strain enhancement of transistor mobility [1] or the transition temperature in ferromagnets [2, 3], ferroelectrics [4], and superconductors [5] have been achieved. Even more exciting is when the epitaxial strain reveals a hidden ground state, such as ferromagnetism [6], ferroelectricity [6, 7], and superconductivity [8] that are absent in the unstrained bulk form of that material system.

Thin films of ferroelectric materials play an essential role in high‐density memory devices [9], which makes the ability to control their ferroelectricity of great interest. For practical device applications and fundamental studies, it is equally desirable to tune the ferroelectric transition over a wide temperature range, from above room temperature to cryogenic temperatures, enabling operation in diverse environments and revealing temperature‐dependent phenomena. Strong coupling between strain and ferroelectricity makes epitaxial strain a particularly efficient tuning knob to control electric polarization and ferroelectricity [10, 11].

The efficiency of strain control of ferroelectricity is captured by the strain derivatives of key properties, such as $dT_{c}/d\varepsilon$% and $dP_{r}/d\varepsilon$%, which quantify the change in transition temperature and remanent polarization per unit strain. For example, while the bulk ${\mathrm{SrTiO}}_{3}$ is paraelectric, epitaxially strained ${\mathrm{SrTiO}}_{3}$ films exhibit emergent ferroelectricity with Curie temperatures reaching room temperature [7, 12]. $dT_{c}/d\varepsilon$ and $dP_{r}/d\varepsilon$ are 83K and $9\;\mu C\;{\mathrm{cm}}^{-2}$, respectively, for ${\mathrm{SrTiO}}_{3}$ [13]. ${\mathrm{KTaO}}_{3}$ is a quantum paraelectric, that is close to a polar instability but remains paraelectric down to low temperature. With $dT_{c}/d\varepsilon$ = 361K and $dP_{r}/d\varepsilon$ = $9\;\mu C\;{\mathrm{cm}}^{-2}$, ${\mathrm{KTaO}}_{3}$ is an ideal candidate for epitaxial strain tuning of ferroelectricity despite its cubic ground state [14, 15].

Tunable electric polarization coupled with enhanced spin–orbit coupling, compared to ${\mathrm{SrTiO}}_{3}$ [16], make ${\mathrm{KTaO}}_{3}$ an intriguing candidate for spintronics applications [17]. The discovery of a 2D electron gas (2DEG) [18, 19, 20] and unconventional superconducting states [21, 22, 23] at ${\mathrm{KTaO}}_{3}$ interfaces combined with reports of efficient spin‐to‐charge interconversion [24, 25] mark ${\mathrm{KTaO}}_{3}$ as an ideal model system to explore these phenomena in the presence of switchable spontaneous polarization. For example, theoretical investigations suggest that Rashba spin splitting can be efficiently improved with the application of compressive stress [26]. Finally, recent theoretical and experimental results point to ferroelectric control and enhancement of superconductivity in incipient ferroelectrics [27, 28, 29].

${\mathrm{KTaO}}_{3}$ is primarily studied in bulk due to the lack of high‐quality epitaxial thin films, which are critical for tuning its physical properties and any potential device applications. High‐quality films grown under adsoprtion‐controlled conditions by molecular‐beam epitaxy (MBE) [30] and hybrid pulsed‐laser depostion [31] have recently become available, enabling epitaxial strain engineering of ${\mathrm{KTaO}}_{3}$ and experimental testing of theoretical predictions [32, 33]. Here, we demonstrate the epitaxial stabilization of the ferroelectric tetragonal polymorph of ${\mathrm{KTaO}}_{3}$ by imposing epitaxial strain through commensurate epitaxial growth on various lattice mismatched substrates under in‐plane compressive strain. The emergence of ferroelectricity is supported by DFT and thermodynamic calculations, the atomic‐scale characterization of the polar displacements using HAADF‐STEM, second‐harmonic generation (SHG) rotational anisotropy response suggesting a 4mm point group, and the observation of ferroelectric hysteresis in metalinsulator–metal (MIM) heterostructures.

## 2 Results

### 2.1 Theoretical Considerations on the Formation of a Polar Point Group

${\mathrm{KTaO}}_{3}$ is a cubic perovskite with a lattice constant of $a_{KTO}$ = 3.988 Å [34] at room temperature. The theoretical lattice parameter of cubic perovskite ${\mathrm{KTaO}}_{3}$ with $Pm\bar{3}m$ (#221) symmetry is 3.990 Å, resolved from first‐principles calculations. As the structure is compressively strained to simulate the properties when grown on ${\mathrm{GdScO}}_{3}$ (−0.5%), ${\mathrm{DyScO}}_{3}$ (−0.9%), and ${\mathrm{SrTiO}}_{3}$ (−2.1%), the out‐of‐plane lattice parameter is relaxed for each tetragonal structure with the P4/mmm (space group #123) symmetry. The ferroelectric transition temperature can be calculated by solving the equation $T_{c}$ = max($T_{C}^{1},T_{C}^{2}$) where $T_{C}^{1}$ and $T_{C}^{2}$ are solutions to [35]

(1) $$\alpha_{1}\left(T_{C}^{1}\right)+\Delta \alpha_{1}=\alpha_{1}T_{C}^{1}-\frac{\left(Q_{11}+Q_{12}\right)\varepsilon_{s}}{s_{11}+s_{12}}=0$$

and

(2) $$\alpha_{1}\left(T_{C}^{2}\right)+\Delta \alpha_{3}=\alpha_{1}T_{C}^{2}-\frac{2Q_{12}\varepsilon_{s}}{s_{11}+s_{12}}=0$$

where $\alpha_{1}$ represents the first‐order Landau coefficient, $\Delta \alpha_{1}$ and $\Delta \alpha_{3}$ denote the changes to the $\alpha_{1}$ coefficient due to strain along the in‐plane and out‐of‐plane directions, respectively, $Q_{11}$ and $Q_{12}$ are components of the electrostrictive tensor and $s_{11}$, $s_{12}$ are the components of the elastic compliance tensor, $\varepsilon_{s}$ is the misfit strain, and $T_{C}^{1}$ and $T_{C}^{2}$ are the transition temperatures due to the compressive and tensile strain, respectively. This allows us to construct the phase transition boundaries between the paraelectric and ferroelectric phases in the temperature–strain phase diagram marked by the dotted lines as illustrated in Figure 1. The dotted boundaries are calculated using a single set of coefficients (Tables S2 and S3). An extended derivation can be found in the Supporting Information II.

**FIGURE 1:**
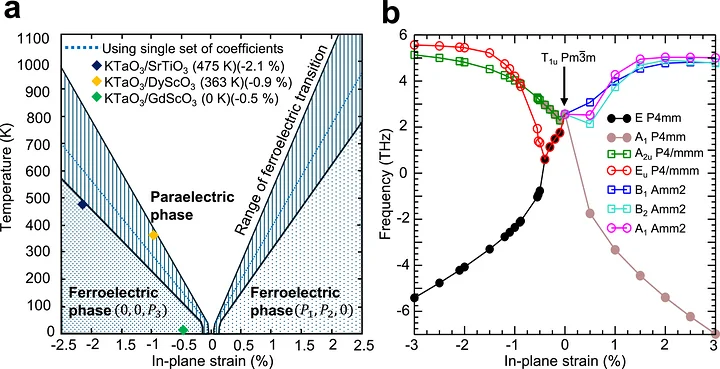
Thermodynamic and DFT investigation of the epitaxial strain control of ferroelectricity in ${\mathrm{KTaO}}_{3}$. (a) Expected shift in $T_{c}$ of (001) ${\mathrm{KTaO}}_{3}$, under epitaxial strain. The experimental values of $T_{c}$ are from SHG experiments (Figure 4 and Figure S13). The components of the spontaneous polarization are given with respect to the pseudocubic axes of ${\mathrm{KTaO}}_{3}$. (b) Lowest phonon frequencies of the structures with the P4/mmm, P4mm, and Amm2 symmetries as a function of epitaxial strain, with imaginary phonon frequencies presented as negative. Tetragonal structure with the P4/mmm symmetry starts to have imaginary phonon frequency beyond 0.5% compressive (tensile) strain with the $A_{2u}$ ($E_{u}$) symmetry. Freezing in this phonon mode leads to a ferroelectric phase transition to a structure with the P4mm (Amm2) symmetry.

The paraelectric (4/mmm) phase in the figure has zero net polarization, that is, $P_{1}=P_{2}=P_{3}=0$. The ferroelectric phases are the tetragonal (4mm) phase with $P_{1}=P_{2}=0$, $P_{3}\neq 0$ and out‐of‐plane polarization, induced by a compressive in‐plane stress, and the orthorhombic (mm2) phase $P_{1}=P_{2}=0$, $P_{3}\neq 0$ showing in‐plane polarization due to the tensile in‐plane stress.

Phonon calculations of the tetragonal structures with the P4/mmm symmetry reveal a Γ‐point instability with the $A_{2u}$ symmetry and $\Gamma_{3}^{-}\left(a\right)$ irreducible representation, with eigenfrequency of 0.94i, 2.57i, and 4.22i THz, for each compressive strain, respectively. When the displacements of the atoms along this phonon frequency with ω=4.22i are frozen into the tetragonal atomic structure, the symmetry is reduced to P4mm (#99). This space group is energetically favored by 14 meV per formula unit compared to the parent P4/mmm structure, for growth on ${\mathrm{SrTiO}}_{3}$ with −2.1% compressive strain. The out‐of‐plane phonon frequency shows a significant hardening from the $A_{2u}$ mode of the P4/mmm structure to the $A_{1}$ mode of the P4mm structure, indicating a second‐order phase transition to the ferroelectric P4mm structure upon compressive strain. The in‐plane phonon frequency with the E symmetry is not significantly affected with increasing compressive strain. Upon application of tensile strain, P4/mmm symmetry structure has a Γ‐point instability with the $E_{u}$ symmetry and $\Gamma_{5}^{-}\left(a,-a\right)$ irreducible representation. At 0.5% tensile strain, this phonon mode is already hardened with a phase transition to the tetragonal ferroelectric structure with the Amm2 symmetry and lowest phonon frequencies of $A_{1}$, $B_{1}$, and $B_{2}$ are presented in Figure 1. The full band diagram of unstrained ${\mathrm{KTaO}}_{3}$ (Figure S1) and under −2.1 % strain (Figure S2) can be found in the Supporting Information Section I. The exact strain value that this transition occurs strongly depends on the exchange and correlational functional choice of DFT. Here, we choose the PBEsol functional because it is stable at the $Pm\bar{3}m$ structure, in agreement with the literature [36], allowing us to study the effects of strain separately, while the SCAN functional is not stable and quantum anharmonic effects play a significant role in ${\mathrm{KTaO}}_{3}$, which is beyond the scope of this manuscript [37, 38].

### 2.2 Growth and Structural Characterization

${\mathrm{KTaO}}_{3}$ grows “cube‐on‐cube” on ${\mathrm{SrTiO}}_{3}$ (001) substrates and “cube‐on‐pseudocube” on ${\mathrm{DyScO}}_{3}$ (110)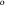 (pseudo‐cubic lattice‐constant 3.947 Å [39], where the subscript O indicates orthorhombic indices) and ${\mathrm{GdScO}}_{3}$ (110)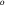 (pseudo‐cubic lattice‐constant 3.967Å [39]). The MBE growth details are described elsewhere [30]. Here, all the films are grown in an adsorption‐controlled regime with a K:Ta excess flux ratio of about 10:1. X‐ray diffraction (XRD) was used to characterize the structural quality of grown films. XRD of the ${\mathrm{KTaO}}_{3}$/${\mathrm{SrTiO}}_{3}$ film is shown in Figure 2a; the ${\mathrm{KTaO}}_{3}$/${\mathrm{DyScO}}_{3}$ and ${\mathrm{KTaO}}_{3}$/${\mathrm{GdScO}}_{3}$ samples are shown in the Supporting Information (Figure S3). The θ–2θ scan shows only 001 peaks, confirming that the film is single‐phase and oriented with its *c*‐axis perpendicular to the plane of the substrate.

**FIGURE 2:**
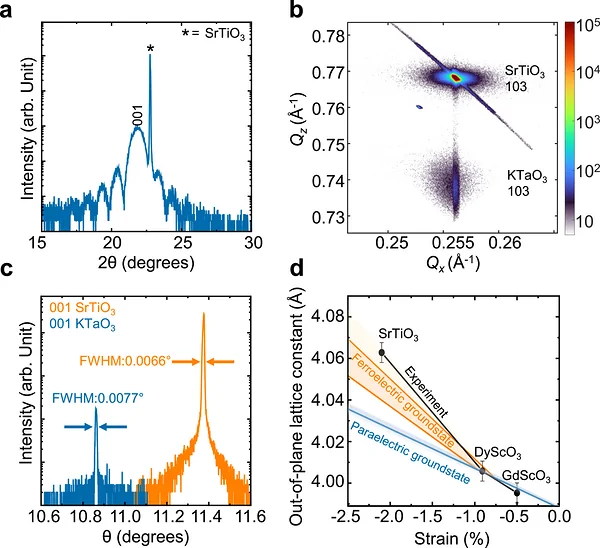
X‐Ray diffraction of a 9.3 nm thick film of ${\mathrm{KTaO}}_{3}$ grown on ${\mathrm{SrTiO}}_{3}$ (001). (a) θ–2θ scan showing the 001 reflection of ${\mathrm{KTaO}}_{3}$ and ${\mathrm{SrTiO}}_{3}$. Symmetric Laue fringes indicate a well‐defined film thickness, indicative of an abrupt interface between film and substrate (asterisks * denotes substrate reflections). (b) X‐ray reciprocal space mapping of the same 9.3 nm thick ${\mathrm{KTaO}}_{3}$ film, confirming that the film is commensurately strained to the ${\mathrm{SrTiO}}_{3}$ substrate. (c) Overlaid rocking curves of the 001 ${\mathrm{KTaO}}_{3}$ and ${\mathrm{SrTiO}}_{3}$ peaks, showing comparable FWHMs, indicating low out‐of‐plane mosaicity (Δω≈ 0.008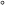). (d) Measured out‐of‐plane lattice constant of ${\mathrm{KTaO}}_{3}$ thin films on various substrates against the expected lattice constants from thermodynamic analysis of the paraelectric (blue) and the ferroelectric (orange) ground states. The range in the calculated out‐of‐plane lattice constant is due to the range in the reported sets of coefficients (Tables S2 and S3).

X‐ray reciprocal space mapping (RSM) around ${\mathrm{SrTiO}}_{3}$ and ${\mathrm{KTaO}}_{3}$ 103 reflections confirm that the films are coherently strained to the substrate (Figure 2b). The full width at half maximum (FWHM) of the rocking curve of the ${\mathrm{KTaO}}_{3}$ (Δω≈ 0.008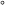) film is as narrow as the ${\mathrm{SrTiO}}_{3}$ substrate (Δω≈ 0.007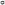), suggesting high crystalline quality of the grown layers (Figure 2c). The films grown on ${\mathrm{GdScO}}_{3}$ and ${\mathrm{DyScO}}_{3}$ both show comparable FWHM values along both orthogonal in‐plane directions of the substrate (Figures S5 and S6).

Thermodynamic analysis was applied to compare the out‐of‐plane lattice constant *c* 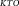 of commensurately strained ${\mathrm{KTaO}}_{3}$ films on different mismatched substrates, using the paraelectric and ferroelectric ground states of ${\mathrm{KTaO}}_{3}$ (Figure 2d). The solid lines are calculated using a single set of coefficients (Tables S2 and S3). The out‐of‐plane lattice constant of the strained ${\mathrm{KTaO}}_{3}$ films were determined from the 00l (l=1,2,3) reflection peaks and the full diffraction spectra, described in the Supporting Information  III. The calculated out‐of‐plane lattice constant, expected for a commensurately strained ${\mathrm{KTaO}}_{3}$ thin film on ${\mathrm{SrTiO}}_{3}$ at room temperature is 4.028 Å, assuming a paraelectric ground state. XRD resolves an out‐of‐plane lattice constant of 4.063 ± 0.005 Å  for the ${\mathrm{KTaO}}_{3}$ film, grown commensurate to the ${\mathrm{SrTiO}}_{3}$ (001) substrate. The discrepancy between the measured and calculated out‐of‐plane lattice parameters could be due to a structural phase transition with epitaxial strain. Figure 2d plots the calculated ferroelectric contribution to the out‐of‐plane lattice constant (orange region). Here, the measured out‐of‐plane lattice constant is consistent with the upper bound of the calculated ferroelectric contribution. The out‐of‐plane lattice constant becomes similar to the calculated value for the paraelectric ground state in partially relaxed films [30]. We also note a previous effort to grow ${\mathrm{KTaO}}_{3}$ films commensurate to ${\mathrm{SrTiO}}_{3}$ (001) using pulsed‐laser epitaxy, reporting a 4.008 ± 0.0005 Å out‐of‐plane lattice constant for ${\mathrm{KTaO}}_{3}$ films [33]. Our results differ significantly from the previous study. The improved composition and structural perfection, achieved through adsorption‐controlled growth, suggest that our results reflect the intrinsic behavior of ${\mathrm{KTaO}}_{3}$.

In contrast to the extended out‐of‐plane lattice spacing observed for the commensurately strained ${\mathrm{KTaO}}_{3}$ films grown on a ${\mathrm{SrTiO}}_{3}$ substrate, the 18 nm thick ${\mathrm{KTaO}}_{3}$ film on ${\mathrm{DyScO}}_{3}$ and the 18 nm thick film on ${\mathrm{GdScO}}_{3}$ appear consistent with the thermodynamic predictions for a paraelectric phase. Atomic force microscopy (AFM) micrographs (Figure S4) reveal a root‐mean‐square (rms) roughness for the 9.3 nm thick ${\mathrm{KTaO}}_{3}$ on ${\mathrm{SrTiO}}_{3}$ of ≈ 0.8 nm, measured by taking a $5\;\mu m^{2}$ area as a reference. For the ${\mathrm{KTaO}}_{3}$ thin films grown on ${\mathrm{DyScO}}_{3}$ and ${\mathrm{GdScO}}_{3}$, the rms are 0.9 and 0.6 nm, respectively (see Figures S5 and S6).

HAADF‐STEM was used to further investigate the polar distortions in the ${\mathrm{KTaO}}_{3}$ films. Figure 3a,b displays HAADF‐STEM images of the ${\mathrm{KTaO}}_{3}$ film grown on a ${\mathrm{SrTiO}}_{3}$ substrate. The interface between ${\mathrm{KTaO}}_{3}$ and ${\mathrm{SrTiO}}_{3}$ is sharp, and the film remains coherently strained to the substrate, indicating a high‐quality epitaxial relationship between the two materials. Figure 3a also includes a zoomed‐in region of the ${\mathrm{KTaO}}_{3}$ film, presented in false color to highlight the displacement of the K column relative to the surrounding Ta columns. The accompanying schematic illustrates the definition of the displacement vector. The displacement vector is defined as the position of the Gaussian‐fitted K‐column relative to the centroid of the four Gaussian‐fitted neighboring Ta‐column positions, $d=r_{K}-r_{Ta-centroid}$. Displacements along the [001] direction (*d* 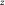) and [100] direction (*d* 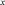) were extracted to quantify the local polar distortion. Figure 3c shows the resulting displacement vector field overlaid on the HAADF‐STEM image. Polar displacements are negligible in the ${\mathrm{SrTiO}}_{3}$ substrate, whereas significant displacements are observed in the ${\mathrm{KTaO}}_{3}$ film, which we attribute to strain‐induced polarization. For a more robust analysis, we examined over 20 000 columns from multiple samples. Figure 3d presents the pooled distribution of displacements along the [001] and [100] crystallographic directions, revealing that the displacements are predominantly aligned along [001], consistent with a preferential out‐of‐plane polarization as predicted theoretically (Figure 1). Figure 3e shows the polar histogram of displacement directions, further confirming that the polar displacements are primarily aligned along [001]. Additional two‐dimensional statistical analysis of the pooled displacement distributions for ${\mathrm{KTaO}}_{3}$ films grown on ${\mathrm{SrTiO}}_{3}$, ${\mathrm{DyScO}}_{3}$, and ${\mathrm{GdScO}}_{3}$, as well as for the ${\mathrm{DyScO}}_{3}$‐based switched metal–insulator–metal (MIM) heterostructure, is provided in Supporting Information Section V. While the film on ${\mathrm{DyScO}}_{3}$ (Figure S9) shows a slight net out‐of‐plane polar displacement, the film on ${\mathrm{GdScO}}_{3}$ (Figure S11) shows no net out‐of‐plane displacement, with average $d_{z}=-1.2$ pm, but does show a finite in‐plane mean displacement, with average $d_{x}=-12.3$ pm. We distinguish this finite in‐plane displacement from the strain‐induced out‐of‐plane tetragonal polar state discussed here, as described in Supporting Information Section V. In addition, HAADF‐STEM performed on the ${\mathrm{DyScO}}_{3}$‐based epitaxial MIM heterostructure that exhibits ferroelectric switching reveals a clear nonzero mean out‐of‐plane displacement in the active device region, consistent with a predominantly out‐of‐plane polar state (Figure S10). Figure 3f exhibits the distribution of the K‐column displacement magnitude. The average magnitude of the polar displacements is 54.08 ± 19.75 pm. This distribution highlights the significant atomic displacements occurring in the ${\mathrm{KTaO}}_{3}$ film due to epitaxial strain. For small distortions, the change in polarization is linear in the atomic displacements. We estimate the spontaneous polarization (*P* 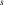) using the following relationship:

(3) $$P_{s}=\frac{e}{V_{\mathrm{KTO}}}\sum_{i}Z_{i}^{*}\times u_{i},$$

where e is the elementary charge, $V_{KTO}$ is the unit cell volume of ${\mathrm{KTaO}}_{3}$, $Z_{i}^{*}$ the Born‐effective charge (BEC) tensor coefficient and $u_{i}$ is the polar atomic distortions. The calculated BEC tensor can be found in Supporting Information VI. Equation (3) sums the contributions of all the ions in the unit cell to spontaneous polarization. Here, only considering the polar distortions from potassium columns, resolved from room temperature HAADF‐STEM, estimates the spontaneous polarization of $P_{s}$ = 18 ± $6\;\mu C\;{\mathrm{cm}}^{-2}$.

**FIGURE 3:**
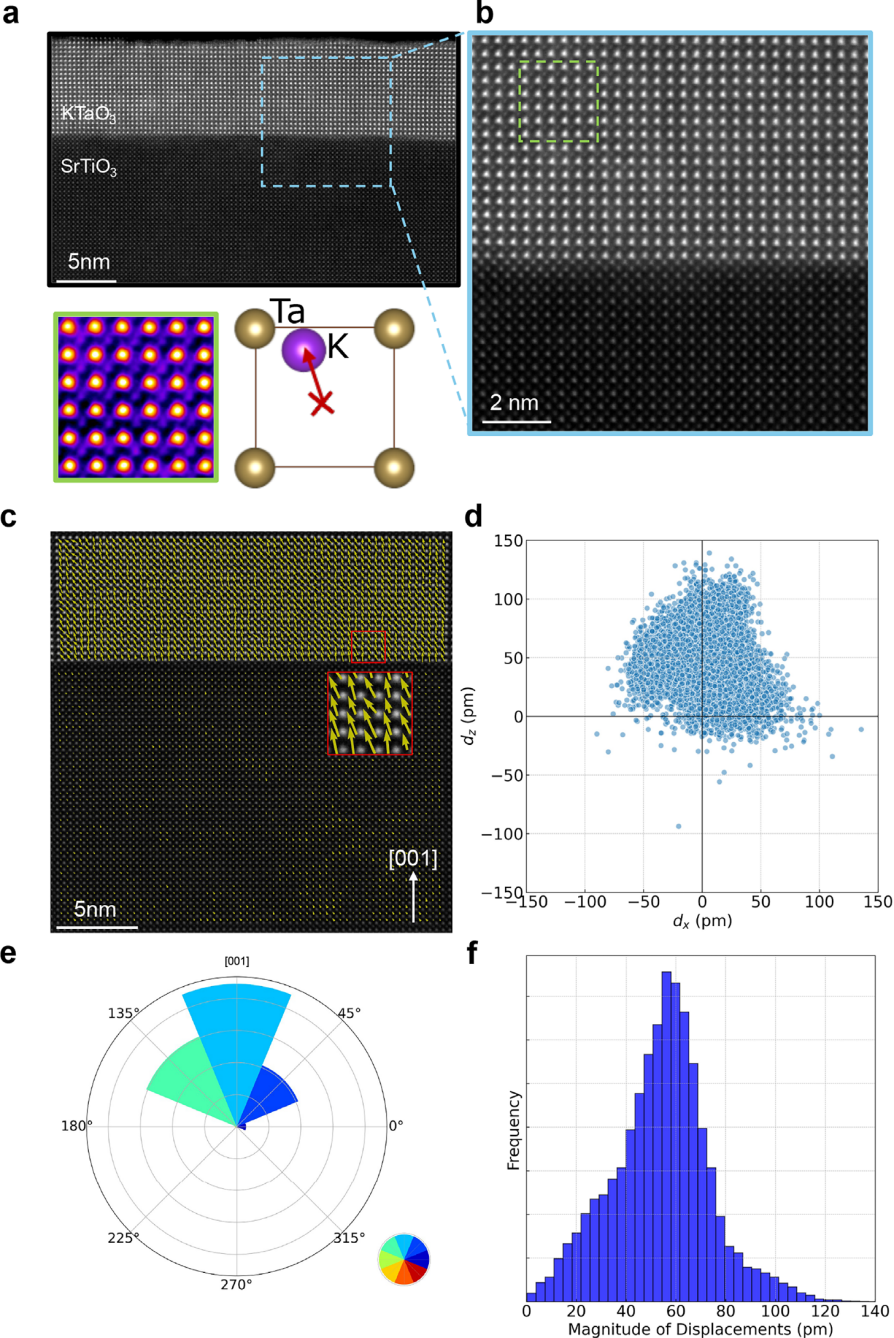
Probing the polar distortions at the atomic scale in epitaxially strained ${\mathrm{KTaO}}_{3}$. (a) HAADF‐STEM image of the ${\mathrm{KTaO}}_{3}$ film grown on ${\mathrm{SrTiO}}_{3}$(001). (b) The interface structure is uniform throughout the sample, indicating a coherent strain between the film and the substrate. No extended defects are observed, which suggests high‐quality epitaxial growth. (c) Displacement vectors of K columns relative to the centroid of the four neighboring Ta columns, overlaid on the HAADF‐STEM image. The K and Ta column positions are obtained by 2D Gaussian fitting, and arrow lengths are scaled for visibility. (d) Tracing of the magnitude and direction of the displacements of atomic columns. (e) Polar histogram, based on more then 20 000 analyzed columns, shows the overall direction of displacements for the ${\mathrm{KTaO}}_{3}$ thin film grown on a ${\mathrm{SrTiO}}_{3}$ substrate. (f) Histogram of the magnitude of the polar displacements in potassium columns, based on the 20 000 columns analyzed.

### 2.3 Probing the Emergence of Ferroelectricity

SHG measurements as a function of temperature were performed for the previously described ${\mathrm{KTaO}}_{3}$/${\mathrm{SrTiO}}_{3}$, ${\mathrm{KTaO}}_{3}$/${\mathrm{DyScO}}_{3}$, and ${\mathrm{KTaO}}_{3}$/${\mathrm{GdScO}}_{3}$ samples. A schematic of the SHG setup used for the experiment is illustrated in Figure 4a. SHG detects a signal that exceeds that of ${\mathrm{SrTiO}}_{3}$ substrate, indicating the long‐range breaking of inversion symmetry. By rotating the polarization of the incident beam, polar plots can be generated, corresponding to *p‐* and *s*‐polarized SHG light reflected off the sample. The derivation of the fitting model is further elaborated in the Supporting Information VII. For the largest in‐plane compressive strain ${\mathrm{KTaO}}_{3}$/${\mathrm{SrTiO}}_{3}$ sample (−2.1%) the phase transition temperature is at 475 K, while for the ${\mathrm{KTaO}}_{3}$/${\mathrm{DyScO}}_{3}$ (−0.9%) samples a polar phase is detected below 360 K. For temperatures below $T_{C}$, the strained ${\mathrm{KTaO}}_{3}$ films display the same tetragonal average symmetry, as shown in Figure 4b, suggesting the absence of any other symmetry‐lowering phase transitions.

**FIGURE 4:**
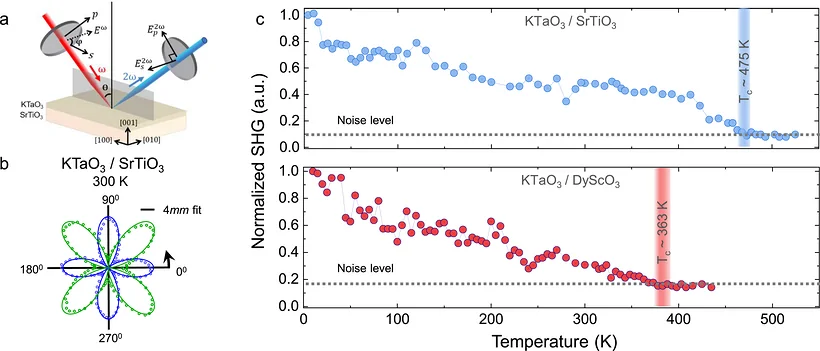
Emergence of the polar P4mm space group in epitaxially strained ${\mathrm{KTaO}}_{3}$. (a) Schematic of SHG setup in reflection geometry. (b) Polar plots of SHG intensity (radius) versus fundamental polarization (azimuth). (c) Normal and oblique incidence SHG intensity with temperature (10–550 K), shown for the 9.3 nm thick ${\mathrm{KTaO}}_{3}$ film grown on ${\mathrm{SrTiO}}_{3}$ and an 18 nm‐thick ${\mathrm{KTaO}}_{3}$ film grown on ${\mathrm{DyScO}}_{3}$.

If ${\mathrm{KTaO}}_{3}$ films on ${\mathrm{SrTiO}}_{3}$ exceed the critical thickness, resulting in strain relaxation, no SHG intensity is detected beyond that of the substrate (see Supporting Information VIII). Rotational anisotropy SHG results are successfully fitted to the 4mm point group, further solidifying the emergence of a polar phase (Figure 4b), consistent with HAADF‐STEM and XRD measurements. Although SHG confirms the emergence of spontaneous electric polarization, it is necessary to exhibit switching of polarization to confirm ferroelectricity.

We note the discrepancy between thermodynamic calculations and experimental evidence, XRD (Figure 2d), HAADF‐STEM (Figure S11), and SHG (Figure 4c) for the ${\mathrm{KTaO}}_{3}$ films grown on ${\mathrm{GdScO}}_{3}$. Here, thermodynamic calculations suggest the emergence of a polar phase at around 100 K, while SHG does not show signal at any temperatures down to 4 K. To further support this observation, SHG intensity was measured as a function of incident beam fluence, which shows no measurable difference between ${\mathrm{KTaO}}_{3}$/${\mathrm{GdScO}}_{3}$ and a bare ${\mathrm{GdScO}}_{3}$ substrate at room and low temperatures (Figure S13c). The out‐of‐plane lattice parameter from XRD (Figure 2d) and the out‐of‐plane displacement extracted from HAADF‐STEM (Figure S11) also show no out‐of‐plane polar order in ${\mathrm{KTaO}}_{3}$/${\mathrm{GdScO}}_{3}$ heterostructure at room temperature. These results suggest that a threshold strain must be applied to stabilize the polar phase. DFT calculations also support a critical epitaxial strain for emergence of ferroelectricity (Figure 1b). The DFT‐resolved critical epitaxial strain for ferroelectric transition, however, depends on the exchange and correlational functional choices and could only qualitatively support the critical strain for ferroelectric transition.

Figure 5a shows phase‐field simulation results, estimating the spontaneous polarization in ${\mathrm{KTaO}}_{3}$ with strain and temperature. Under epitaxial strain of –2.1%, as imposed by coherent growth on ${\mathrm{SrTiO}}_{3}$, the spontaneous polarization reaches ∼ $26\;\mu C\;{\mathrm{cm}}^{-2}$ at liquid helium temperature. The predicted room temperature electric polarization is $∼19\;\mu C\;{\mathrm{cm}}^{-2}$, which is consistent with the STEM‐resolved polarization (18 ± $6\;\mu C\;{\mathrm{cm}}^{-2}$) using Equation (3). We note reports of oxygen octahedra distortions under strain [40] and potassium desorption [41] in ${\mathrm{KTaO}}_{3}$. Furthermore, significant anion contributions to spontaneous polarization is reported in ${\mathrm{NaNbO}}_{3}$ [42]. Here, the cation polar distortions, unlike ${\mathrm{NaNbO}}_{3}$, are significant and their contribution alone could explain the calculated spontaneous polarization, suggesting that anion contributions to electric polarization are negligible.

**FIGURE 5:**
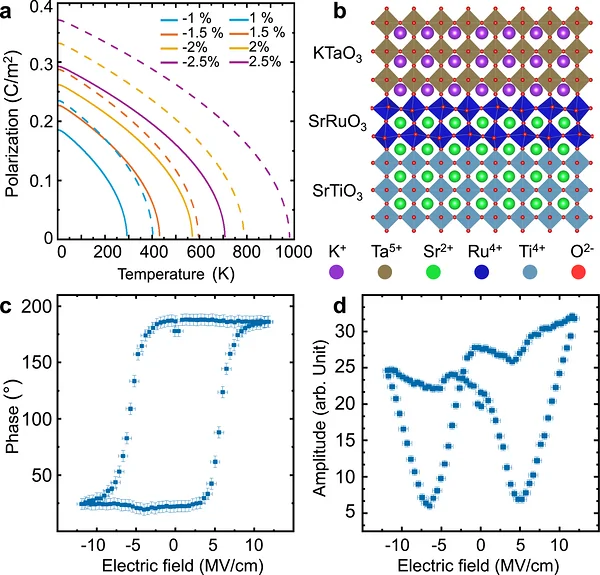
Switching of spontaneous electric polarization in epitaxially strained ${\mathrm{KTaO}}_{3}$. (a) Thermodynamic calculation of the polarization as a function of epitaxial strain and temperature. (b) Schematic of the epitaxial heterostructure used for PFM measurements. (c) PFM‐resolved hysteresis loop of ${\mathrm{KTaO}}_{3}$/${\mathrm{SrRuO}}_{3}$/${\mathrm{SrTiO}}_{3}$ heterostructure, confirming electric switching of spontaneous electric polarization. (d) Magnitude of the piezo response.

To determine whether this strain‐induced electric polarization is switchable under applied electric field, a capacitor stack was fabricated: ${\mathrm{KTaO}}_{3}$ grown on a conductive ${\mathrm{SrRuO}}_{3}$ bottom electrode on ${\mathrm{SrTiO}}_{3}$ (001). For structural details, see Supporting Information III. Local electromechanical measurements using piezoresponse force microscopy (PFM) were used to reveal ferroelectric switching and the hysteresis loop, instead of conventional PUND techniques, due to the leakage current that often dominates electrical measurements in ultrathin layers [43]. Figure 5b illustrates the heterostructure used for PFM: ${\mathrm{KTaO}}_{3}$/${\mathrm{SrRuO}}_{3}$/${\mathrm{SrTiO}}_{3}$, where ${\mathrm{SrRuO}}_{3}$ serves as an epitaxial bottom electrode. Clear 180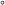 phase reversal for out‐of‐plane PFM response at an arbitrary location on the capacitors of ${\mathrm{KTaO}}_{3}$/${\mathrm{SrRuO}}_{3}$/${\mathrm{SrTiO}}_{3}$ is shown in Figure 5c. At a quasi‐static frequency of 1.1 Hz standard ferroelectric $d_{33}-V$ is observed with coercive voltages of ± 5.7 V. The error bars represent the average response over the 10 cycles of a “pulse‐mode” triangular wave. The butterfly‐shaped amplitude curve confirms ferroelectric switching (Figure 5d). Additionally, ${\mathrm{KTaO}}_{3}$/${\mathrm{SrRuO}}_{3}$/${\mathrm{DyScO}}_{3}$ capacitor heterostructure was investigated using PFM. A 180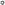 phase reversal for the out‐of‐plane PFM response is observed (Figure S14). This result is consistent with thermodynamic calculations (Figure 1a) and SHG (Figure 4b), exhibiting an above room temperature polar transition. The ${\mathrm{KTaO}}_{3}$ films grown on ${\mathrm{DyScO}}_{3}$, however, do not demonstrate an extended XRD lattice constant (Figure 2d) and HAADF‐STEM shows a small net out‐of‐plane polar distortion (Figure S9). This could be due to the presence of polar nano‐regions and an order–disorder ferroelectric transition, which has been reported in strained ${\mathrm{SrTiO}}_{3}$ films [44, 45, 46]. The switched MIM test structures commensurate to ${\mathrm{DyScO}}_{3}$, on the other hand, exhibit a significant enhancement of out‐of‐plane polar distortion in HAADF‐STEM compared to the film grown directly on ${\mathrm{DyScO}}_{3}$ (Figure S10). Finally, we do not observe any polar switching behavior in similar heterostructures grown on ${\mathrm{GdScO}}_{3}$.

In conclusion, high‐quality ${\mathrm{KTaO}}_{3}$ thin films were coherently strained to various substrates exerting a compressive in‐plane strain ranging from ‐0.5% on ${\mathrm{GdScO}}_{3}$ up to −2.1% on ${\mathrm{SrTiO}}_{3}$. The emergence of a tetragonal polar P4mm phase is predicted by DFT and thermodynamic calculations and experimentally confirmed by diffraction, SHG, and HAADF‐STEM. We demonstrated the switching of the emergent spontaneous electric polarization using PFM. The room temperature phase‐field spontaneous polarization (19 μC ${\mathrm{cm}}^{-2}$) is consistent with the experimentally resolved value (18 ± 6 μC ${\mathrm{cm}}^{-2}$) using HAADF‐STEM atomic polar distortions and calculated BEC tensor. We have thus revealed a ferroelectric ground state in epitaxially strained ${\mathrm{KTaO}}_{3}$.

Given the recent discovery of unconventional superconductivity at ${\text{KTaO}}_{3}$ interfaces [21, 47, 48] and reports of ferroelectric enhancement of superconductivity [28, 29, 49], tunable ferroelectricity in ${\mathrm{KTaO}}_{3}$ provides a platform for evaluating theoretical proposals that link superconductivity to a proximal ferroelectric state [27, 50, 51, 52].

## 3 Methods

### 3.1 Thin Film Synthesis and Characterization

Epitaxial ${\mathrm{KTaO}}_{3}$ films were grown in a modified Vecco GEN 10 MBE system. The substrate was heated using a 10 μm ${\mathrm{CO}}_{2}$‐laser, Epiray GmbH (THERMALAS Substrate Heater). Films were grown by co‐deposition of potassium, tantalum, and ozone at a substrate temperature of 650 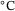, measured using an optical pyrometer operating at a 7.5μm wavelength. Here, all the films were grown in an adsorption‐controlled regime with a K:Ta flux ratio of approximately 10:1. A mixture of ozone and oxygen (10% $O_{3}$ + 90% $O_{2}$) was used as the oxidant. The films were grown at an oxidant background pressure of $1\times 10^{-6}$ Torr. Typical fluxes for the sources were $\left(4-7\right)\times 10^{12}$ ${\text{atoms/cm}}^{2}/s$ for the tantalum source and $\left(4-7\right)\times 10^{13}$ ${\text{atoms/cm}}^{2}/s$ for the potassium source, determined by a quartz crystal microbalance (QCM), with an accuracy of about ± 15%. Co‐deposition with these fluxes results in a ${\mathrm{KTaO}}_{3}$ film growth rate of about 0.03 Å/s. Additional MBE growth details are described elsewhere [30].

X‐ray diffraction (XRD), x‐ray reflectometry (XRR), and reciprocal space mapping (RSM) measurements were carried out using a PANalytical Empyrean diffractometer with Cu $K\alpha_{1}$ radiation. The raw XRR spectra were analyzed using the PANalytical X'Pert Reflectivity software package and the layer thickness was derived from a fast Fourier transform (FFT) after manually defining the critical angle to account for refractive effects. In situ reflection high‐energy electron diffraction (RHEED) patterns were recorded using kSA‐400 software and a Staib electron source operated at 14 kV and a filament current of 1.5 A. The morphology of the film surface was characterized using an Asylum Cypher ES environmental AFM. Piezoforce microscopy (PFM) was conducted on an Asylum Cypher AFM using Dual AC Resonance Tracking (DART) mode.

### 3.2 Transmission Electron Microcopy

Cross‐sectional scanning transmission electron microscopy (STEM) samples were prepared using a standard lift‐out process with a Thermo Fisher Scientific Helios G4 UX focused ion beam, with a final milling voltage of 2 kV for the Ga ions. Atomic‐resolution HAADF‐STEM images of the ${\mathrm{KTaO}}_{3}$/${\mathrm{SrTiO}}_{3}$, ${\mathrm{KTaO}}_{3}$/${\mathrm{DyScO}}_{3}$, and ${\mathrm{KTaO}}_{3}$/${\mathrm{GdScO}}_{3}$ samples were acquired on a Thermo Fisher Scientific Spectra 300 X‐CFEG operated at 200 kV with a convergence angle of 30 mrad and a HAADF detector collection range of 60–200 mrad. Additional cross‐sectional STEM imaging of the ${\mathrm{DyScO}}_{3}$‐based capacitor heterostructure was performed at the Center for Electron Microscopy and Analysis (CEMAS) at The Ohio State University using a Thermo Fisher Scientific Themis Z microscope operated at 200 kV with a probe semi‐convergence angle of 20 mrad and a HAADF detector collection range of 64–200 mrad. To improve signal‐to‐noise ratio, 25 fast‐scan images (2048 × 2048 pixels, 200 ns dwell time) were aligned and averaged. Atomic column positions were first identified automatically using Atomap, followed by nearest‐neighbor assignment. The positions were then refined in two steps: first by center‐of‐mass refinement and subsequently by 2D Gaussian fitting of the column intensities. The displacement of each K column was then calculated relative to the centroid of the four neighboring Ta columns.

### 3.3 Second‐Harmonic Generation

Second‐harmonic generation (SHG) measurements were made with an 800 nm fundamental wavelength pulsed laser line from a Spectra‐Physics Solstice Ace laser focused on the sample, while second‐harmonic light generated at 400 nm was measured reflecting off the samples. The repetition rate of the laser was 1 kHz, while the beam size at focus was 16 μm. All measurements were done in reflection geometry. For low temperature measurements, a Janis 30 gas flow cryostat was used, while for high‐temperature measurements, a homemade heater was employed.

### 3.4 Density Functional Theory

The atomic structure relaxation, phonon, and Berry phase calculations were performed using first‐principles density functional theory within the generalized gradient approximation (GGA) as implemented in the PBEsol functional [53] by the Vienna Ab initio Simulation Package (VASP) [54] with the ion–electron interaction described by the projector augmented wave method [55] including the spin‐orbit coupling (SOC) effects. We employed an energy cutoff of 700 eV, Gaussian smearing of 0.005 eV, and electronic energy tolerance of $10^{-8}$ eV for the total energy convergence. The ionic relaxations were performed with a force tolerance of $10^{-3}$ Å/eV and an electronic momentum *k*‐point mesh of 24×24×24. Phonon and Berry phase calculations were performed with an electronic momentum *k*‐point mesh of 16×16×16. Phonon calculations were analyzed using the Phonopy code [56].

### 3.5 Thermodynamics

Using Landau–Ginzburg–Devonshire theory for ferroelectrics, the elastic Gibbs free energy density *g*, written as a power series of the polarization near the phase transition (Supporting Information Discussion II), describes the ferroelectric phase transition [57]. The ferroelectic transition temperature can be calculated by solving Equations (1) and (2), which allows the construct of the phase transition boundaries between the paraelectric and ferroelectric phases in the temperature‐strain phase diagram. Since there is some variation in the data collected on the electrostrictive coefficients and elastic constants from the literature, we list the values by different groups in Tables S2 and S3 and include the regions indicating the range of ferroelectric transition to account for the spread in these values exhibited in Figure 1a.

## Conflicts of Interest

The authors declare no conflicts of interest.

## Supporting information


**Supporting File**: adma74366‐sup‐0001‐SuppMat.pdf.

## Data Availability

The data that support the findings of this study are available within the article. Additional data related to film growth and structural characterization by XRD and STEM are available at https://doi.org/10.34863/1a2q‐0380.
