# Supplementary material for: Above Room Temperature Ferroelectricity in Epitaxially Strained KTaO3
Source: Adv Mater. 2026 Aug 11;38(57):e74366. doi: 10.1002/adma.74366 (PMC13660824; doi:10.1002/adma.74366)
Supplement: Supplementary file 1 [file ADMA-38-e74366-s001.pdf]

Above Room Temperature Ferroelectricity in Epitaxially Strained $KTaO_3$

Tobias Schwaigert,[1,2,a)] Salva Salmani-Rezaie,[3,4,a)] Sankalpa Hazra,[5,a)] Utkarsh Saha,[5] Maya Ramesh,[2] Aiden Ross,[5] Betül Pamuk,[6] Long-Qing Chen,[5] David A. Muller,[3,4] Darrell G. Schlom,[1,2,4,7] Venkatraman Gopalan,[5,8] and Kaveh Ahadi[9,10,b)]

[1)] *Platform for the Accelerated Realization, Analysis, and Discovery of Interface Materials (PARADIM), Cornell University, Ithaca, New York 14853, USA*

[2)] *Department of Materials Science and Engineering, Cornell University, Ithaca, NY 14853, USA*

[3)] *School of Applied and Engineering Physics, Cornell University, Ithaca, New York 14853, USA*

[4)] *Kavli Institute at Cornell for Nanoscale Science, Cornell University, Ithaca, New York 14853, USA*

[5)] *Material Science and Engineering, Pennsylvania State University, University Park, PA 16802, USA*

[6)] *Department of Physics and Astronomy, Williams College, Williamstown, Massachusetts 01267, USA*

[7)] *Leibniz-Institut für Kristallzüchtung, Max-Born-Str. 2, 12489 Berlin, Germany*

[8)] *Deparment of Physics, Pennsylvania State University, University Park, PA 168010, USA*

[9)] *Department of Materials Science and Engineering, Ohio State University, Columbus, OH 43210 USA*

[10)] *Department of Electrical and Computer Engineering, Ohio State University, Columbus, OH 43210 USA*

(Dated: 28 July 2026)

Supporting Information: Above Room Temperature Ferroelectricity in Epitaxially Strained $KTaO_3$

[a] Authors contributed equally
[b] ahadi.4@osu.edu

I. PHONON FREQUENCIES AND ENERGY GAINS FROM FIRST PRINCIPLES

The atomic structure relaxation, phonon, and Berry phase calculations were performed using first-principles density functional theory within the generalized gradient approximation (GGA) as implemented in the PBEsol functional[1] by the Vienna Ab initio Simulation Package (VASP)[2] with the ion-electron interaction described by the projector augmented wave method[3] including the spin-orbit coupling (SOC) effects. We employed an energy cut-off of 700 eV, Gaussian smearing of 0.005 eV, and electronic energy tolerance of 10^{-8} eV for the total energy convergence. The ionic relaxations were performed with a force tolerance of 10^{-3} Å/eV and an electronic momentum k-point mesh of $24 \times 24 \times 24$. Phonon and Berry phase calculations were performed with an electronic momentum k-point mesh of $16 \times 16 \times 16$. Phonon calculations were analyzed using the Phonopy code[4].

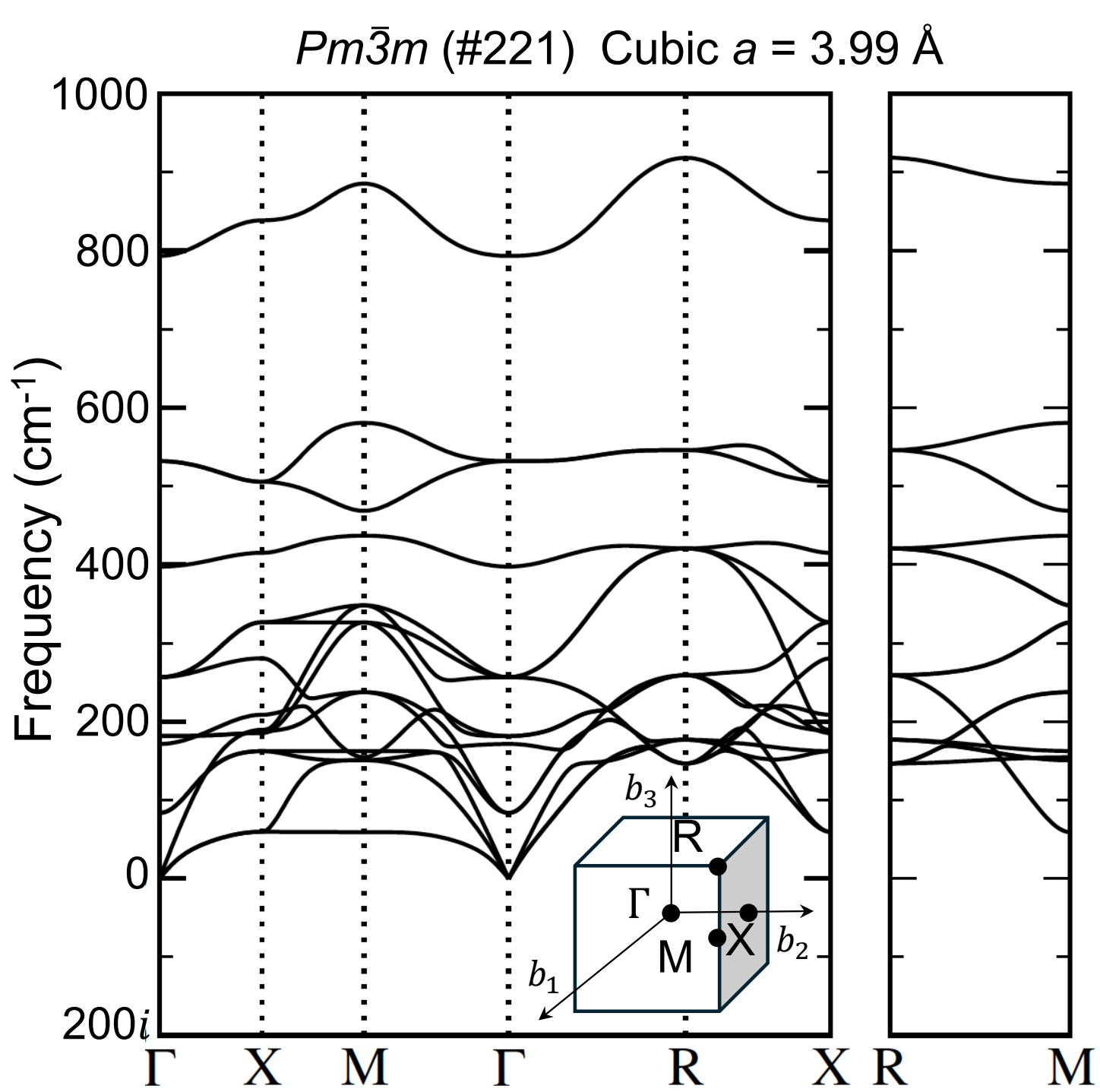

Fig. S1. Phonon band diagram for unstrained $KTaO_3$.

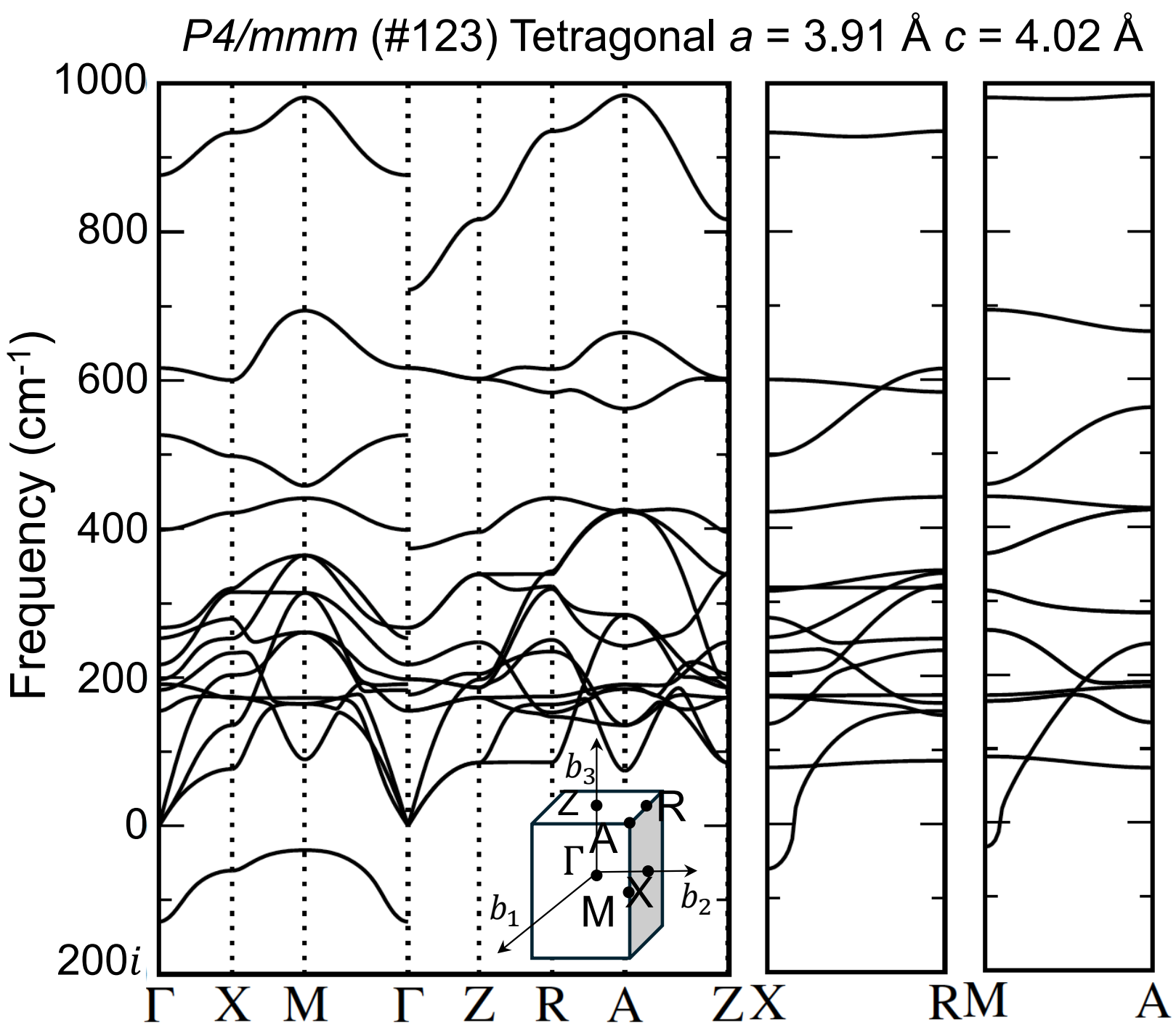

Fig. S2. Phonon band diagram for epitaxial strain conditions on $SrTiO_3$ (-2.1%).

II. DERIVATION OF PHASE FIELD MODEL AND FERROELECTRIC TRANSITION TEMPERATURE

Based on the Landau-Ginzburg-Devonshire theory of ferroelectrics, the Gibbs free energy density g can be written as a power series of the polarization near the phase transition.[5]

$$
\begin{aligned}
g = g(T, P_i, \sigma_i) - g(T, P_i) = 0, \sigma_i = 0 \\
&= \alpha_1(T)(P_1^2 + P_2^2 + P_3^2) + \alpha_{11}(P_1^4 + P_2^4 + P_3^4) + \alpha_{12} \\
&(P_1^2 P_2^2 + P_1^2 P_3^2 + P_2^2 P_3^2) - \frac{1}{2} s_{11}(\sigma_1^2 + \sigma_2^2 + \sigma_3^2) - s_{12} \\
&(\sigma_1\sigma_2 + \sigma_1\sigma_3 + \sigma_3\sigma_2) - \frac{1}{4} s_{44}(\sigma_4^2 + \sigma_5^2 + \sigma_6^2) - Q_{11} \\
&(\sigma_1 P_1^2 + \sigma_2 P_2^2 + \sigma_3 P_3^2) - Q_{12}(\sigma_1(P_2^2 + P_3^2) + \sigma_2(P_1^2 + P_3^2) + \\
&\sigma_3(P_1^2 + P_2^2)) - Q_{44}(P_2 P_3 \sigma_4 + P_1 P_3 \sigma_5 + P_1 P_2 \sigma_6), \quad (1)
\end{aligned}
$$

where P_i represents the ith component of polarization and σ_i represents the ith component of the applied stress in Voigt notation. s_{11}, s_{11}, and s_{44} are the elastic compliance constants of

the cubic phase, and Q_{11}, Q_{12}, and Q_{44} are the electrostrictive coefficients. The rectangular Cartesian frame of reference is used the the x_1, x_2, and x_3 axes along the (100), (010), and (001) crystallographic directions. The Landau expansion coefficients α_1, α_{11}, and α_{12} along with the elastic compliance constants and the elctrostrictive coefficents for the potential function are listed in Table S1.

TABLE S1. Set of coefficients of the potential function

Coefficients	Values
T_O	1.6 K
T_S	18 K
C	6.98 $\times 10^4$ K
$\alpha_1(T)$	$\frac{1}{2\varepsilon_c C}\left(T_S \coth\left(\frac{T_S}{T}\right) - T_o\right)$ C^{-2} m^6 N
α_{11}	3 $\times 10^9$ C^{-4} m^6 N
α_{12}	3.3 $\times 10^9$ C^{-4} m^6 N
s_{11}	2.555 $\times 10^{-12}$ Pa^{-1}
s_{12}	-0.493 $\times 10^{-12}$ Pa^{-1}
s_{44}	9.174 $\times 10^{-12}$ Pa^{-1}
Q_{11}	0.0872 C^{-2} m^4
Q_{12}	-0.023 C^{-2} m^4
Q_{44}	0.0297 C^{-2} m^4

To investigate the effect of strain on the phase transitions of $KTaO_3$ thin films epitaxially grown on a substrate, we use the thin film boundary conditions and the modified free energy f for the strained thin film case.[6]

$$f = \alpha_1^* \left(P_1^2 + P_1^2\right) + \alpha_3^* P_3^2 + \alpha_{11}^* \left(P_1^4 + P_1^4\right) + \alpha_{33}^* P_3^4 + \alpha_{11}^* \left(P_1^2 P_3^2 + P_2^2 P_3^2\right) + \alpha_{12}^* \left(P_1^2 P_2^2\right) + \frac{\epsilon_s^2}{s_{11} + s_{12}} \tag{2}$$

with

$$\alpha_1^* = \alpha_1 - \frac{\left(Q_{11} + Q_{12}\right)\epsilon_s}{s_{11} + s_{12}} \tag{3}$$

$$\alpha_3^* = \alpha_1 - \frac{2Q_{12}\epsilon_s}{s_{11} + s_{12}} \tag{4}$$

$$\alpha_3^* = \alpha_{11} - \frac{\left(Q_{11}^2 + Q_{12}^2\right) s_{11} - 2Q_{11}Q_{12}s_{11}}{2\left(s_{11}^2 - s_{12}^2\right)} \tag{5}$$

$$\alpha_{33}^* = \alpha_{11} + \frac{Q_{12}^2}{s_{11} + s_{12}} \tag{6}$$

$$\alpha_{12}^* = \alpha_{12} - \frac{1}{s_{11}^2 - s_{12}^2}\left[\left(Q_{11}^2 + Q_{12}^2\right) s_{12} - 2Q_{11}Q_{12}s_{11}\right] + \frac{Q_{44}^2}{2s_{44}} \tag{7}$$

$$\alpha_{13}^* = \alpha_{12} + \frac{Q_{12}\left(Q_{11} + Q_{12}\right)}{s_{11} + s_{12}} \tag{8}$$

where ϵ_s represents the misfit strain and in this case, $\epsilon_s = \frac{a_{sub} - a_o}{a_{sub}}$ where a_{sub} is the lattice constant of the substrate and a_o is the lattice constant of the unstrained cubic film.

The ferroelectic transition temperature can be calculated by solving the equation $T_c = \max(T_C^1, T_C^2)$ where T_C^1 and T_C^2 are solutions to

$$\alpha_1\left(T_C^1\right) + \Delta\alpha_1 = \alpha_1 T_C^1 - \frac{\left(Q_{11} + Q_{12}\right)\epsilon_s}{s_{11} + s_{12}} = 0 \tag{9}$$

and

$$\alpha_1\left(T_C^2\right) + \Delta\alpha_3 = \alpha_1 T_C^2 - \frac{2Q_{12}\epsilon_s}{s_{11} + s_{12}} = 0 \tag{10}$$

which allows us to construct the phase transition boundaries between the paraelectric and ferroelectric phases in the temperature-strain phase diagram marked by the dotted lines as illustrated in Fig. 1a. The dotted boundaries are calculated using the single set of coefficients (See Supplemental discussion and Table S2).

Since there is some variation in the data collected on the electrostrictive coefficients and elastic constants from the literature, we list the values by different groups in Table S2 and S3, and include the regions indicating the range of ferroelectric transition to account for the spread in these values.

TABLE S2. Electrostrictive coefficients in units of C^{-2} m^4

	Ref. 7	Ref 8
Q_{11}	0.087 ± 0.006	0.1256
Q_{12}	-0.023 ± 0.002	-0.0313
Q_{44}	0.0297 ± 0.023	-

TABLE S3. Elastic compliances in units of 10^{-12} Pa^{-1}

	Ref 9	Ref 10	Ref 10	Ref 11
s_{11}	2.555	2.47	2.17	2.36
s_{12}	-0.493	-0.325	-0.254	-0.307
s_{44}	9.174	5.9	5.076	11.69

III. ADDITIONAL SAMPLE CHARACTERIZATION OF $KTaO_3$ FILMS

High-resolution X-ray diffraction (XRD; Panalytical Emperyean) using Cu $K_{\alpha1}$ radiation was performed on $KTaO_3$ thin films grown on different substrates. Figure S3 show θ-2θ scans of phase-pure epitaxial (001)-oriented $KTaO_3$ films on three different substrates ($SrTiO_3$, $DyScO_3$, and $GdScO_3$). In total 9.3 nm of $KTaO_3$ were deposited on $SrTiO_3$ (001) and 18 nm on each of $DyScO_3$ $(110)_o$ and $GdScO_3$ $(110)_o$. The presence of Laue fringes indicate that each film has a well-defined thickness i.e., smooth interfaces between film and substrate and between film and air. These Laue fringes around the thin films peak were used to calculate the thickness and were equal to the thickness extracted from HAADF-STEM. The in-plane lattices constants of the strained $KTaO_3$ films shown in Fig. 2d were determined from the 00ℓ (ℓ=1,2,3) reflection peaks. Supplementary Figure S5a shows the reciprocal space

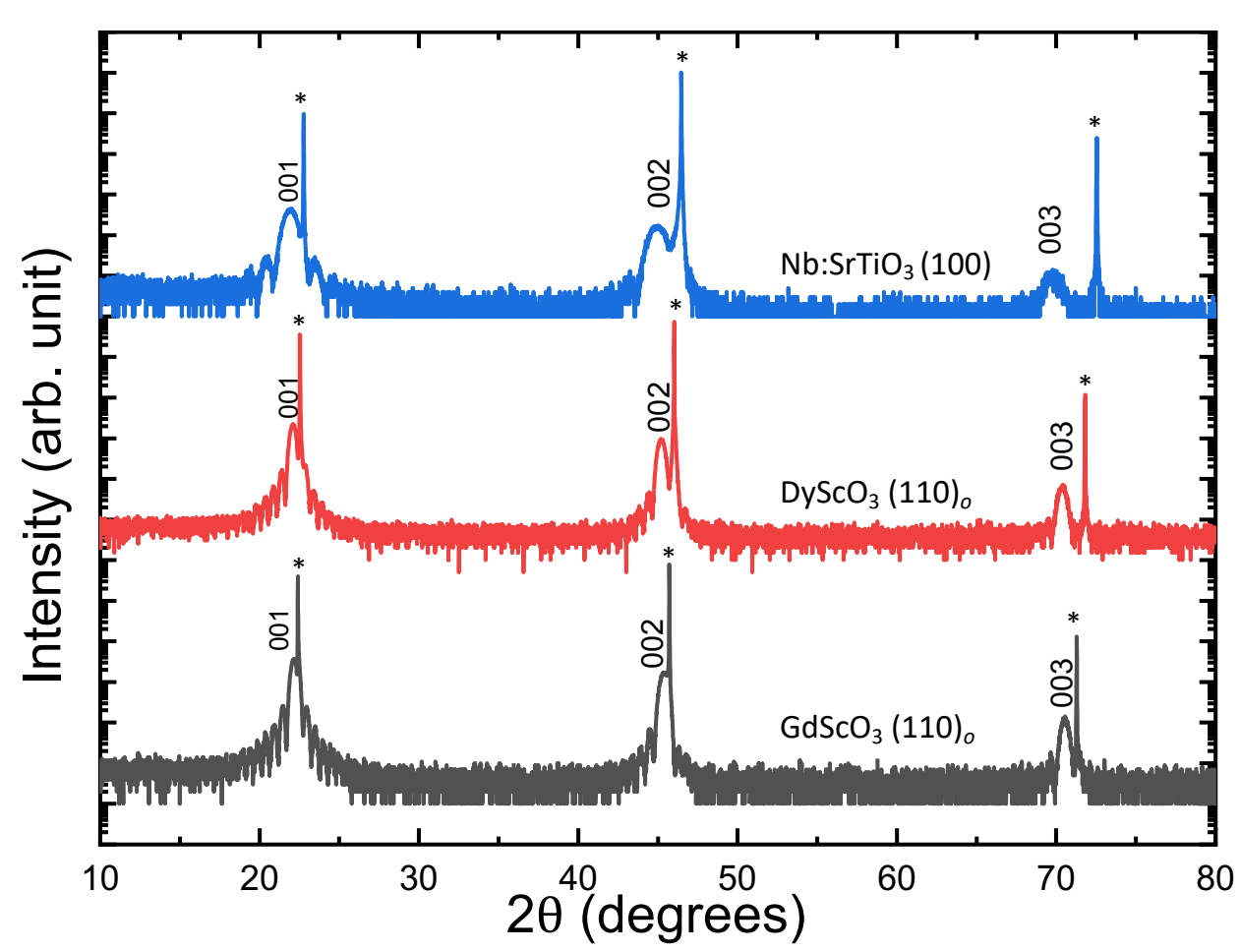

Fig. S3. θ-2θ XRD scan of $KTaO_3$ films on (001) $SrTiO_3$ (blue), $(110)_o$ $DyScO_3$, (red) and $(110)_o$ $GdScO_3$ (grey) substrates.

maps (RSM) around the 103 $KTaO_3$ and 332 $DyScO_3$ $(110)_o$. The film is commensurately

strained to the substrate. The FWHM of the rocking curves of film (0.009°/0.003°) and substrate (0.008°/0.003°) are comparable, suggesting high crystalline quality (Supplementary Fig. S5b,d). The rocking curves were measured along the [001] and $[1\bar{1}0]$ in-plane directions of the $DyScO_3$ substrate, respectively. This is the narrowest FWHM ever reported for a $KTaO_3$ film. Figure S5c,f shows AFM images at different magnifications. The root-mean-square (rms) roughness is 0.8 nm taking a 5 µm^2 area as the reference.

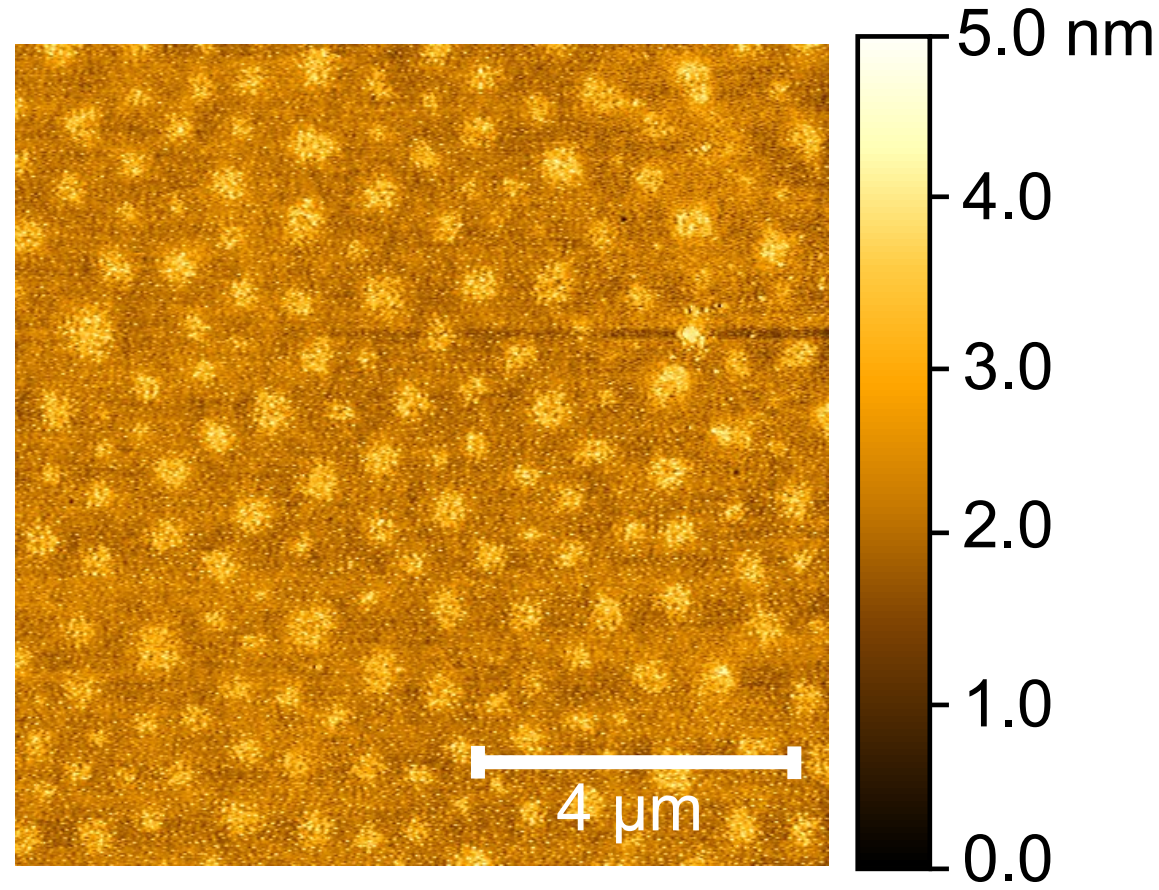

Fig. S4. AFM images of the untreated 9.3 nm thick $KTaO_3$ film shown through Figs. 2-4. This film exhibits an rms roughness of 0.8 nm over the whole 5 µm^2 scan.

Figure S6**a** shows the RSM around the 103 $KTaO_3$ and 332 $GdScO_3$ reflection and suggests that the film is commensurately strained to the substrate. The FWHM of the rocking curves of film (0.009°/0.008°) and substrate (0.017°/0.016°) comparable suggesting high crystalline quality (Fig. S5b,d). Figure S6 shows the AFM images at different magnifications with a rms of 0.6 nm taking a 5 µm^2 area as the reference.

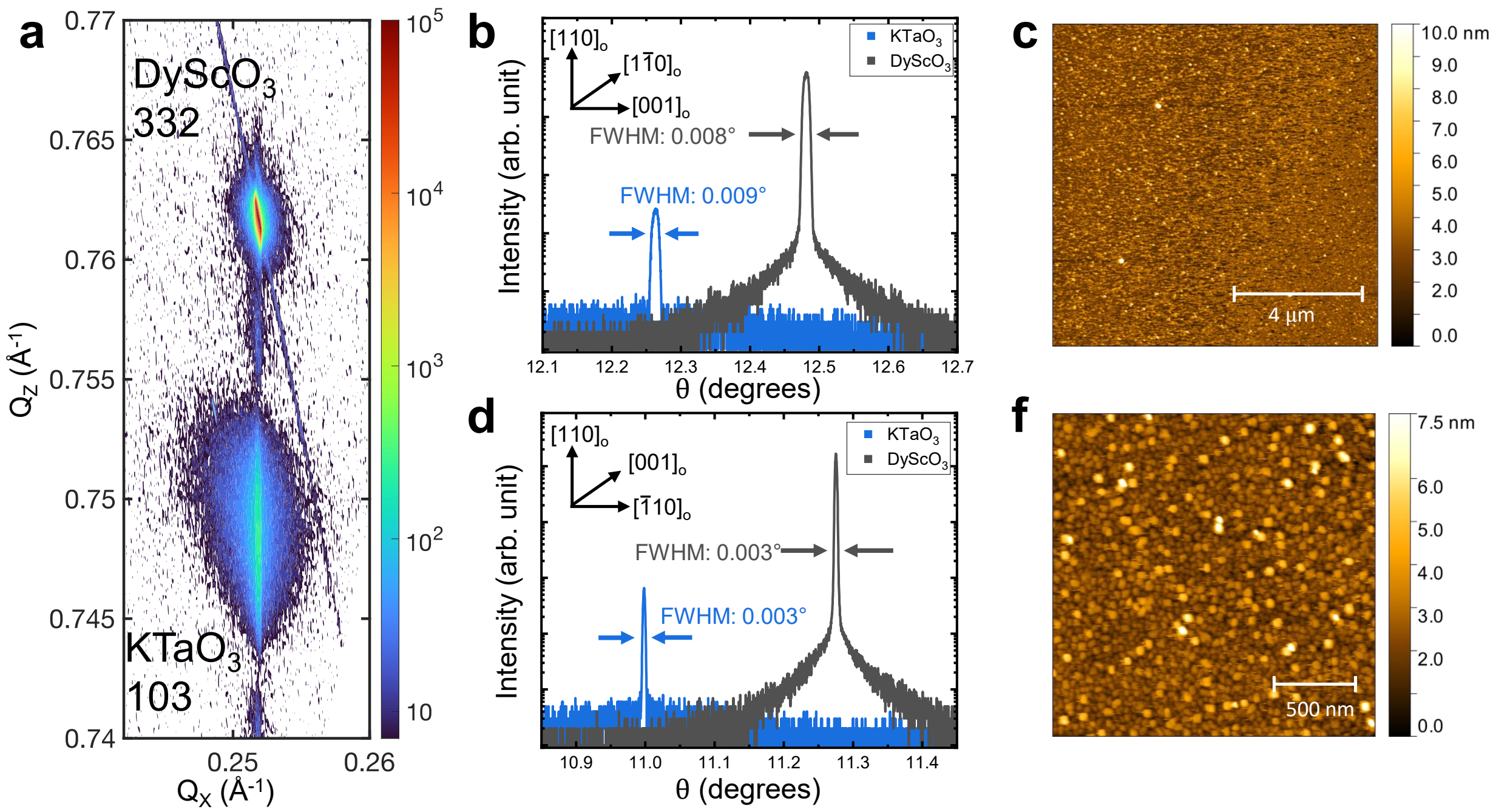

Fig. S5. **18 nm $KTaO_3$ on $DyScO_3$.** **a** RSM of the $KTaO_3$ 103 and $DyScO_3$ 332 peaks. **b and d** Overlaid rocking curves of the $KTaO_3$ 001 and $GdScO_3$ 110 peaks, showing comparable FWHMs, indicating low out-of-plance mosaicity ($\Delta\omega \approx 0.009°$ and $0.003°$ along the two orthogonal in-plane directions of the substrate.) **c,f** AFM images at different magnifications. The root mean square (rms) roughness over the $5\,\mu m^2$ scan is 0.9 nm.

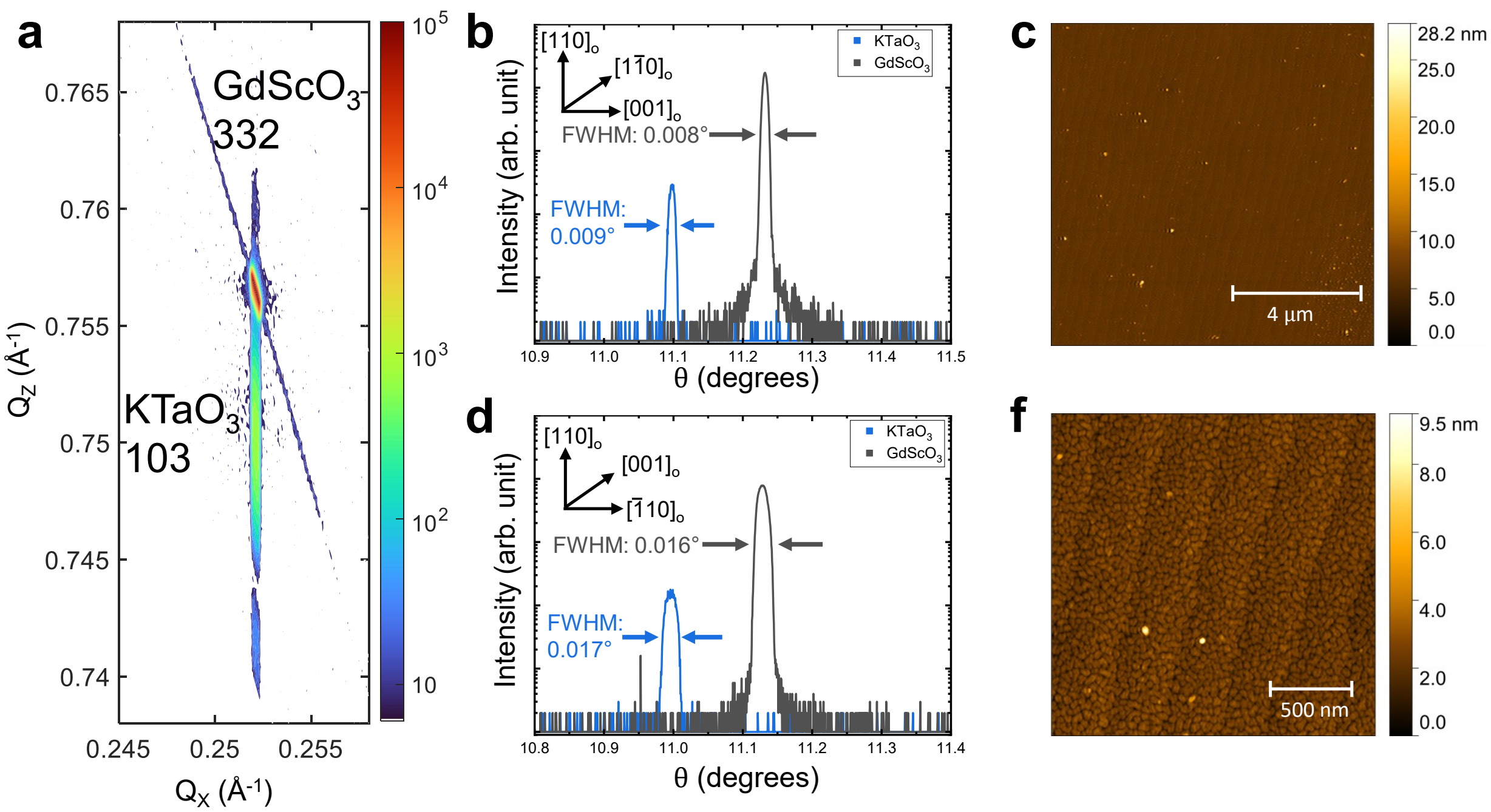

Fig. S6. **18 nm $KTaO_3$ on $GdScO_3$** **a** RSM of the $KTaO_3$ 103 and $GdScO_3$ 332 peaks. **b and d** Overlaid rocking curves of the $KTaO_3$ 001 and $GdScO_3$ 110 peaks, showing comparable FWHMs, indicating low out-of-plance mosaicity ($\Delta\omega \approx 0.009°$ and $0.017°$ along the two orthogonal in-plane directions of the substrate.) **c,f** AFM images at different magnifications. The root mean square (rms) roughness over the $5\,\mu m^2$ scan is 0.6 nm.

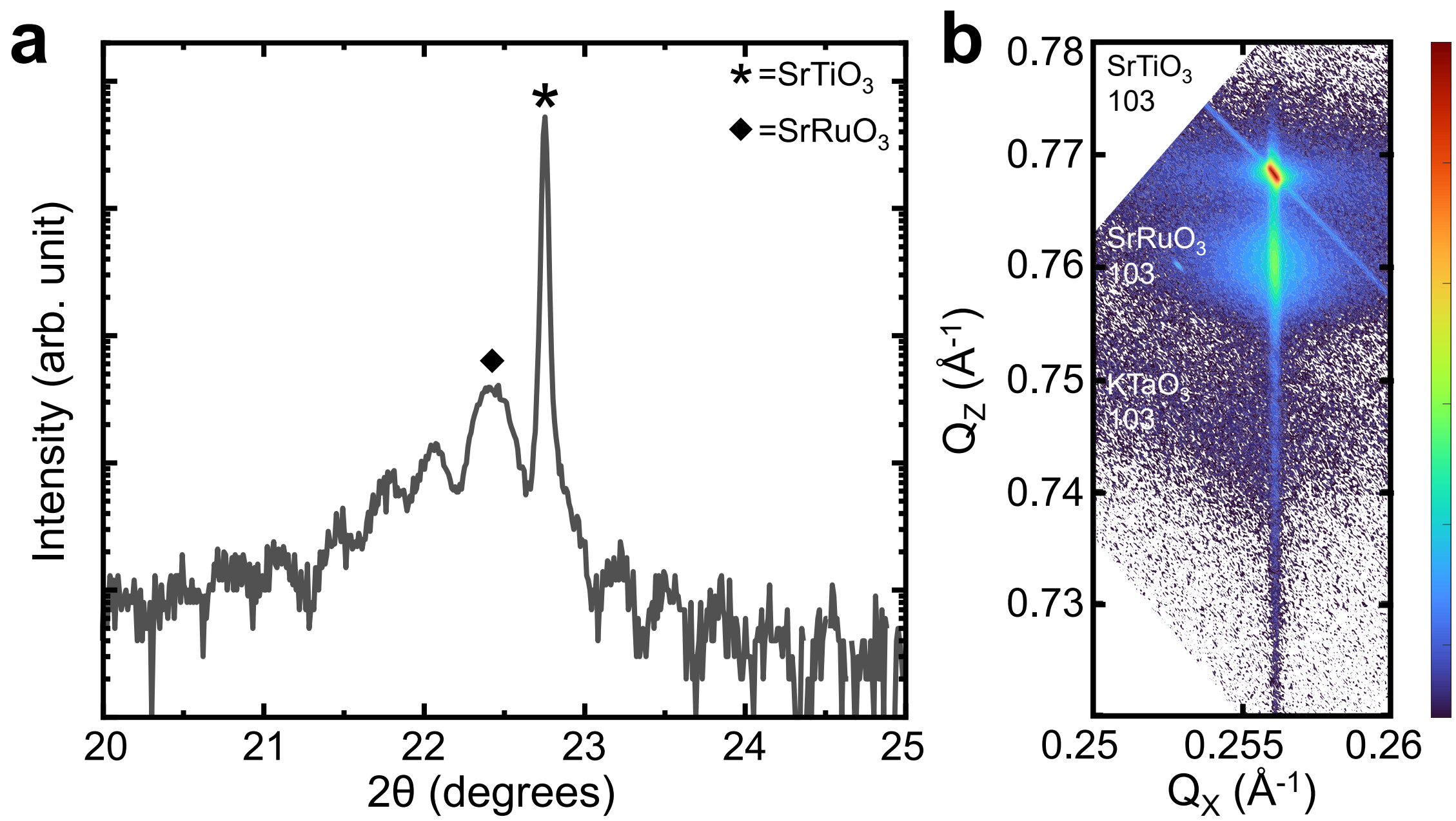

Fig. S7. **a** θ-2θ XRD scan of $KTaO_3$ films on a 35 nm thick bottom electrode $SrRuO_3$ deposited on a $SrTiO_3$ substrate. **b** RSM of the $KTaO_3$ 103, $SrRuO_3$ 332, and $SrTiO_3$ 103 peaks.

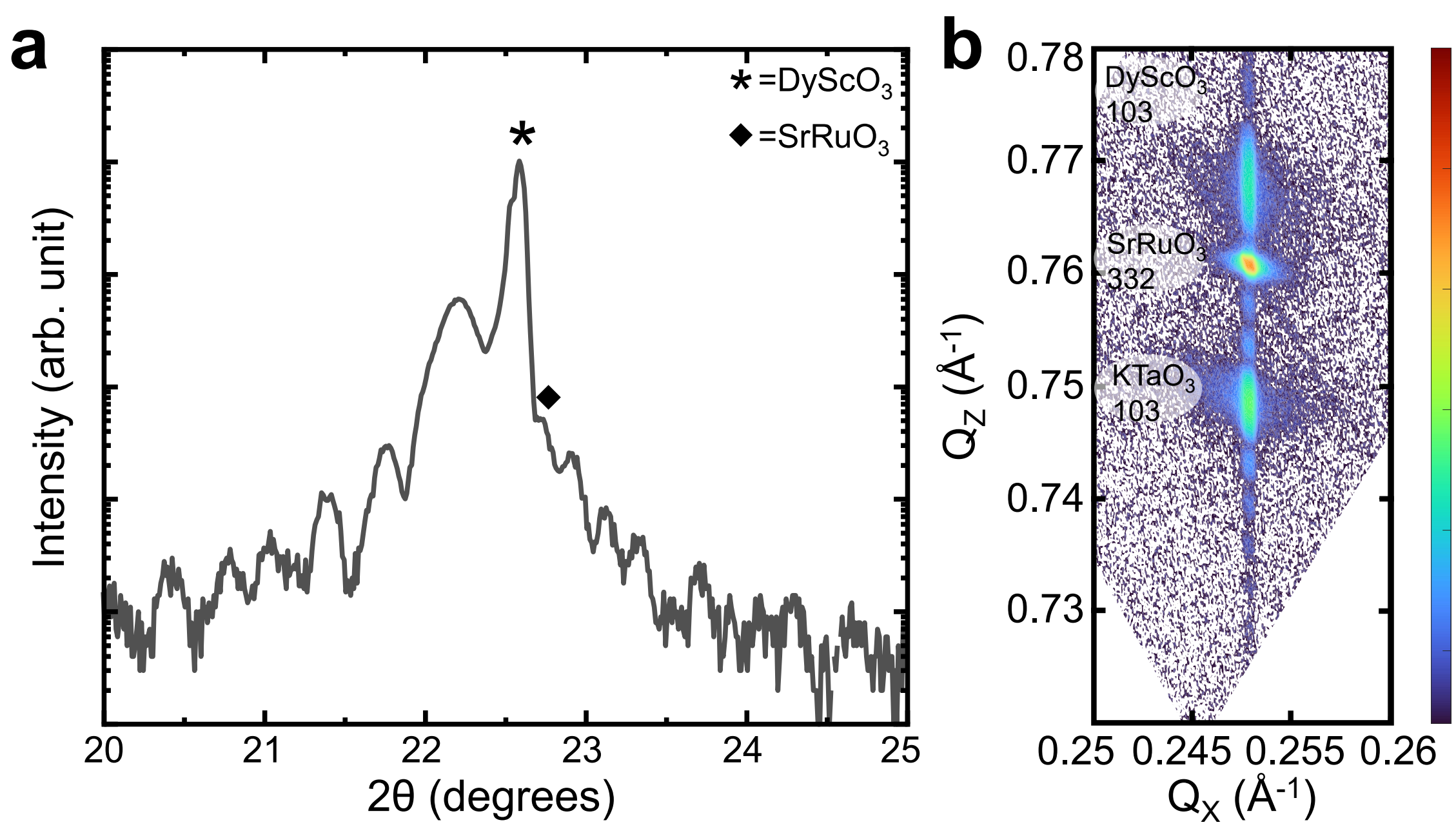

Fig. S8. **a** θ-2θ XRD scan of $KTaO_3$ films on a 20 nm thick bottom electrode $SrRuO_3$ deposited on a $DyScO_3$ substrate. **b** RSM of the $KTaO_3$ 103, $SrRuO_3$ 332, and $DyScO_3$ 332 peaks.

IV. THERMODYNAMIC ANALYSIS OF EFFECT OF STRAIN ON PARA AND FERROELECTRIC GROUND STATE OF $KTAO_3$

For a ferroelectric thin film epitaxially grown on a substrate, we may assume that the in-plane strains are fixed by the lattice parameter of the substrate and the out-of-plane components of stress are free to relax. Therefore, the in-plane components of strain (ε_1 , ε_2), and ε_6) and the out-of-plane components of stress (σ_3 , σ_4, and σ_5) will be independent variables leaving, the in-plane components of stress (σ_1 , σ_2, and σ_6) and the out-of-plane components of strain (ε_3, ε_4, and ε_5) as dependent variables. While equation S1 describes the thermodynamic behavior using stress as the independent variable and strain as the dependent variable, we can transform this equation to be more suitable for ferroelectric thin films,

$$f(T, P, \varepsilon_1, \varepsilon_2, \sigma_3) = u - Ts - \sigma_3\varepsilon_3 = g + \sigma_1\varepsilon_1 + \sigma_2\varepsilon_2 + \sigma_6\varepsilon_6 \tag{11}$$

To perform the Legendre transform, we must find $\sigma_1(T, P, \varepsilon_1, \varepsilon_2, \sigma_3)$, $\sigma_2(T, P, \varepsilon_1, \varepsilon_2, \sigma_3)$ and $\sigma_6(T, P, \varepsilon_1, \varepsilon_2, \sigma_3)$. For simplicity we shall only consider the impact of normal strains. To do this we shall first calculate

$$\varepsilon_1(T, P, \sigma_1, \sigma_2, \sigma_3) = -\left(\frac{\partial g}{\partial \sigma_1}\right)_{T,P} = Q_{11}P_1^2 + Q_{12}(P_2^2 + P_3^2) + S_{11}\sigma_1 + S_{12}(\sigma_2 + \sigma_3) \tag{12}$$

$$\varepsilon_2(T, P, \sigma_1, \sigma_2, \sigma_3) = -\left(\frac{\partial g}{\partial \sigma_2}\right)_{T,P} = Q_{11}P_2^2 + Q_{12}(P_1^2 + P_3^2) + S_{11}\sigma_2 + S_{12}(\sigma_1 + \sigma_3) \tag{13}$$

Then algebraically solve for $\sigma_1(T, P, \varepsilon_1, \varepsilon_2, \sigma_3)$ and $\sigma_2(T, P, \varepsilon_1, \varepsilon_2, \sigma_3)$ which yields

$$\sigma_1 = \frac{\varepsilon_1 - (Q_{11}P_1^2 + Q_{12}(P_2^2 + P_3^2)) - (S_{12} - \frac{S_{12}^2}{S_{11}})\sigma_3 + \frac{S_{12}}{S_{11}}(Q_{11}P_2^2 + Q_{12}(P_2^2 + P_3^2) - \varepsilon_2)}{S_{11} - \frac{S_{12}^2}{S_{11}}}, \tag{14}$$

$$\sigma_2 = \frac{\varepsilon_2 - (Q_{11}P_2^2 + Q_{12}(P_1^2 + P_3^2)) - (S_{12} - \frac{S_{12}^2}{S_{11}})\sigma_3 + \frac{S_{12}}{S_{11}}(Q_{11}P_1^2 + Q_{12}(P_1^2 + P_3^2) - \varepsilon_1)}{S_{11} - \frac{S_{12}^2}{S_{11}}}. \tag{15}$$

Substituting in these expressions for $\sigma_1(T, P, \varepsilon_1, \varepsilon_2, \sigma_3)$ and $\sigma_2(T, P, \varepsilon_1, \varepsilon_2, \sigma_3)$ in equation 2.1 we obtain an expression that uses the in-plane strain and the out-of-plane stress as the independent variables.

$$f(T,P,\varepsilon_1,\varepsilon_2,\sigma_3) = g(T,P,\sigma_1(T,P,\varepsilon_1,\varepsilon_2,\sigma_3),\sigma_1(T,P,\varepsilon_1,\varepsilon_2,\sigma_3),\sigma_3)+\sigma_1(T,P,\varepsilon_1,\varepsilon_2,\sigma_3)\varepsilon_1+\sigma_2(T,P,\varepsilon_1,\varepsilon_2,\sigma_3)\varepsilon_2 \tag{16}$$

Where the out-of plane strain is found by taking the derivative of the free-energy with respect to the out-of plane stress

$$\varepsilon_3 = \left(\frac{\partial f}{\partial \sigma_3}\right)_{T,P,\varepsilon_1,\varepsilon_2} \tag{17}$$

Assuming we have a tetragonal C domain $P_i = (0,0,P_3)$, we can find that the out-of plane strain is

$$\varepsilon_3 = \frac{(\varepsilon_1+\varepsilon_2)S_{12}}{S_{11}+S_{12}} + \left(Q_{11} - \frac{2Q_{12}S_{12}}{S_{11}+S_{12}}\right)P_3^2 \tag{18}$$

Then, to obtain the lattice parameter one can use the relation

$$\varepsilon_3 = \frac{c-a^{eq}}{a^{eq}}, \tag{19}$$

where $a^{eq}$is the equivalent cubic lattice parameter of a stress-free, polarization-free, cubic $KTaO_3$. Combining equation 20 and 21 we find,

$$c = a^{eq}[1+\frac{(\varepsilon_1+\varepsilon_2)S_{12}}{S_{11}+S_{12}} + (Q_{11} - \frac{2Q_{12}S_{12}}{S_{11}+S_{12}})P_3^2], \tag{20}$$

In the paraelectric phase, $P_i = (0,0,0)$, which yields

$$c = a^{eq}[1+\frac{(\varepsilon_1+\varepsilon_2)S_{12}}{S_{11}+S_{12}}], \tag{21}$$

and reduces to the solution derived from elasticity theory.

V. STATISTICAL ANALYSIS OF ATOMIC DISPLACEMENTS

The displacement distributions shown in Figs. S9-S12 were compiled from multiple HAADF-STEM images acquired from several analyzed regions of each sample. For each region, the positions of the K columns and the four neighboring Ta columns were obtained by 2D Gaussian fitting, and the displacement of the K column relative to the centroid of the surrounding Ta columns was extracted. The resulting pooled distributions are used to

compare the net polar distortions in $KTaO_3$ films grown on different substrates. For $KTaO_3$ grown on $DyScO_3$, the pooled displacement distribution compiled from multiple HAADF-STEM images and analyzed regions shows a slight net out-of-plane bias. The extracted mean values are $\langle d_x \rangle$= -1.6 pm and $\langle d_z \rangle$= 4.6 pm, indicating a weak preferential displacement along the out-of-plane [001] direction. This result is consistent with a weak out-of-plane polar distortion in the DyScO3-strained film at room temperature, smaller than that observed for the $SrTiO_3$-strained case. To further clarify the $DyScO_3$ case, we also analyzed a cross-sectional lamella prepared from the same $DyScO_3$-based capacitor heterostructure for which ferroelectric switching is shown in Fig. S14. In this switched device region, the extracted displacement distribution yields $\langle d_x \rangle$= -4.3 pm and $\langle d_z \rangle$= 21.2 pm, with the principal axis oriented primarily along the out-of-plane direction and an aspect ratio of 1.59. This reveals a clear net out-of-plane polar distortion in the active device region. The larger out-of-plane displacement compared with the pooled $DyScO_3$ film data is consistent with spatial heterogeneity of the polar distortion and with the weaker, near-transition character of the $DyScO_3$-strained film. In contrast, for $KTaO_3$ grown coherently on $GdScO_3$, the pooled displacement distribution does not show a net out-of-plane polar distortion. The extracted mean values are $d_x = -12.3$ pm and $d_z = -1.2$ pm, indicating that the average out-of-plane displacement remains centered near $d_z \approx 0$. Here, the finite in-plane mean displacement is not negligible. However, we do not interpret it as evidence for the strain-induced out-of-plane tetragonal polar state discussed in the main text. This distinction is based on the direction of the displacement and its consistency with complementary measurements. The measured mean displacement in $KTaO_3/GdScO_3$ is primarily in-plane, whereas the polar state discussed in the main text is out-of-plane and tetragonal. In addition, the $GdScO_3$ film shows no SHG signal beyond the bare substrate down to 4 K, no extended out-of-plane lattice constant, and no ferroelectric switching. We use "correlated polar distortion" to refer to a spatially coherent displacement pattern that produces a net symmetry breaking detectable by complementary probes such as SHG, XRD, or switching measurements. By this definition, the $GdScO_3$ film does not show a long-range correlated out-of-plane polar state. The finite pooled $\langle d_x \rangle$ is instead consistent with possible local structural heterogeneity and short-range distortions in this weakly strained film, rather than with a coherent net out-of-plane polar state. Figure S12 shows the pooled two-dimensional displacement distribution for $KTaO_3$ grown on $SrTiO_3$. The distribution exhibits a strong net out-of-plane bias,

with $\langle d_x \rangle$= -3.1 pm and $\langle d_z \rangle$= 46.3 pm. Unlike the $DyScO_3$ and $GdScO_3$ cases, the STO distribution is not unimodal, but instead exhibits two dominant populations with opposite in-plane displacements and similar positive out-of-plane components. This indicates that while the average in-plane displacement remains near zero, the out-of-plane polar distortion is robust and common to both local populations. The bimodal distribution therefore reflects locally distinct in-plane offsets superimposed on the same predominantly out-of-plane polar state, rather than a reversal of the out-of-plane polarization. A quantitative summary of the principal-axis-based analysis of the pooled displacement distribution is given in Table S4. Here, $\langle d_x \rangle$ and $\langle d_z \rangle$ describe the average in-plane and out-of-plane displacements, respectively. The principal-axis angle θ gives the overall orientation of the pooled distribution, while σ_{major} and σ_{minor} describe its characteristic widths along the major and minor axes. Their ratio provides a compact measure of the overall elongation of the pooled cloud. Because the STO distribution is bimodal, these quantities should be interpreted only as a summary of the overall shape of the distribution; the contour plot more directly captures the existence of the two local populations. The positive $\langle d_z \rangle$ confirms a strong average out-of-plane polar displacement, while the near-zero $\langle d_x \rangle$ indicates that the opposite in-plane components largely cancel on average. The aspect ratio of 1.30 indicates moderate elongation of the pooled distribution. Together, these parameters provide a compact summary of the overall distribution, while the contour plot in Fig. S12 more clearly reveals the physically important two-lobed structure.

TABLE S4. Quantitative summary of the pooled 2D displacement distribution for $KTaO_3$ grown on $SrTiO_3$.

**Parameter**	**Meaning**	**Value**
$\langle d_x \rangle$ = (pm)	Mean in-plane displacement	-3.1
$\langle d_z \rangle$ = (pm)	Mean out-of-plane displacement	46.3
θ (deg)	Orientation of the principal axis of the distribution	154.5
σ_{major} (pm)	Standard deviation along the major principal axis	27.1
σ_{minor} (pm)	Standard deviation along the minor principal axis	20.8
σ_{major} /σ_{minor}	Aspect ratio of the distribution	1.30

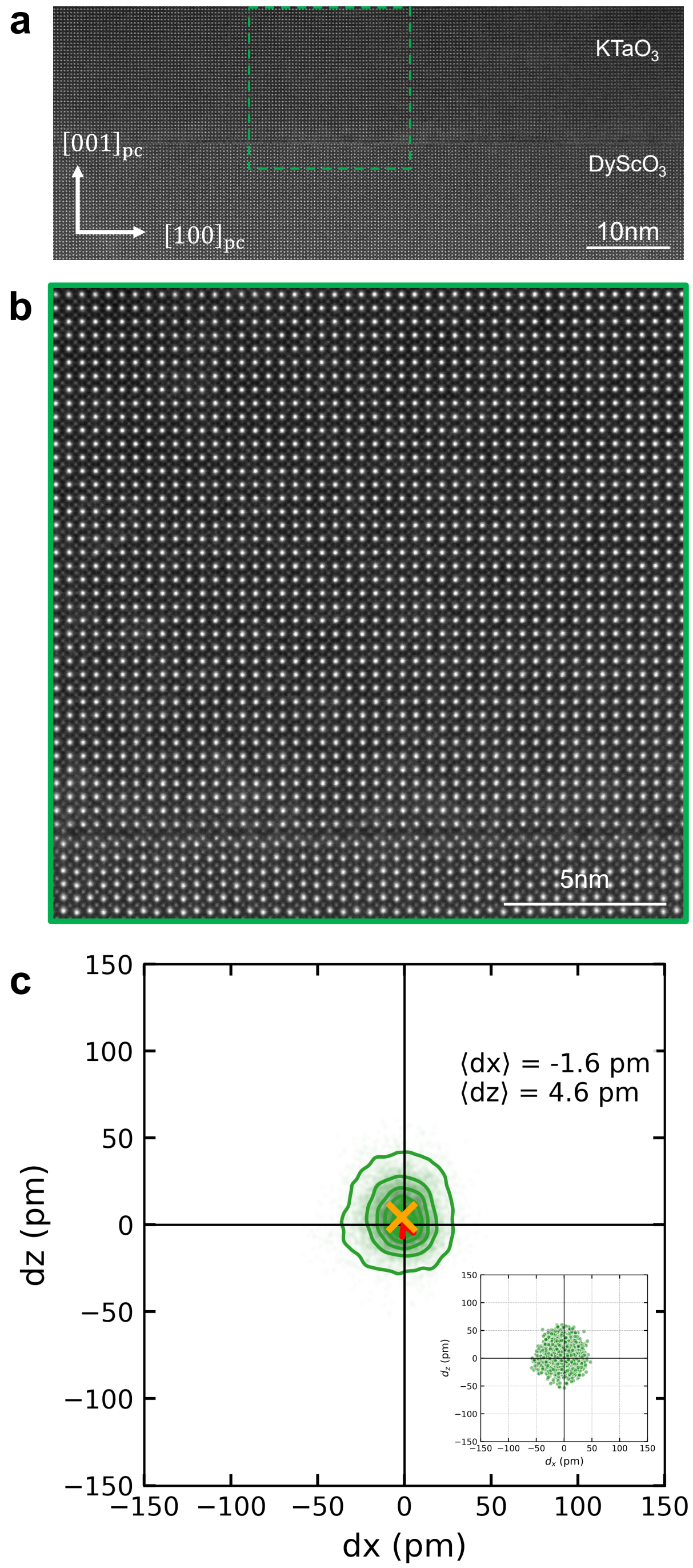

Fig. S9. **a,b** HAADF-STEM images of the $KTaO_3/DyScO_3$ heterostructure. **c** Pooled two-dimensional distribution of K-column displacements relative to the centroid of the four neighboring Ta columns, compiled from multiple HAADF-STEM images acquired from several analyzed regions. The contour lines summarize the distribution, and the orange marker denotes the mean displacement.

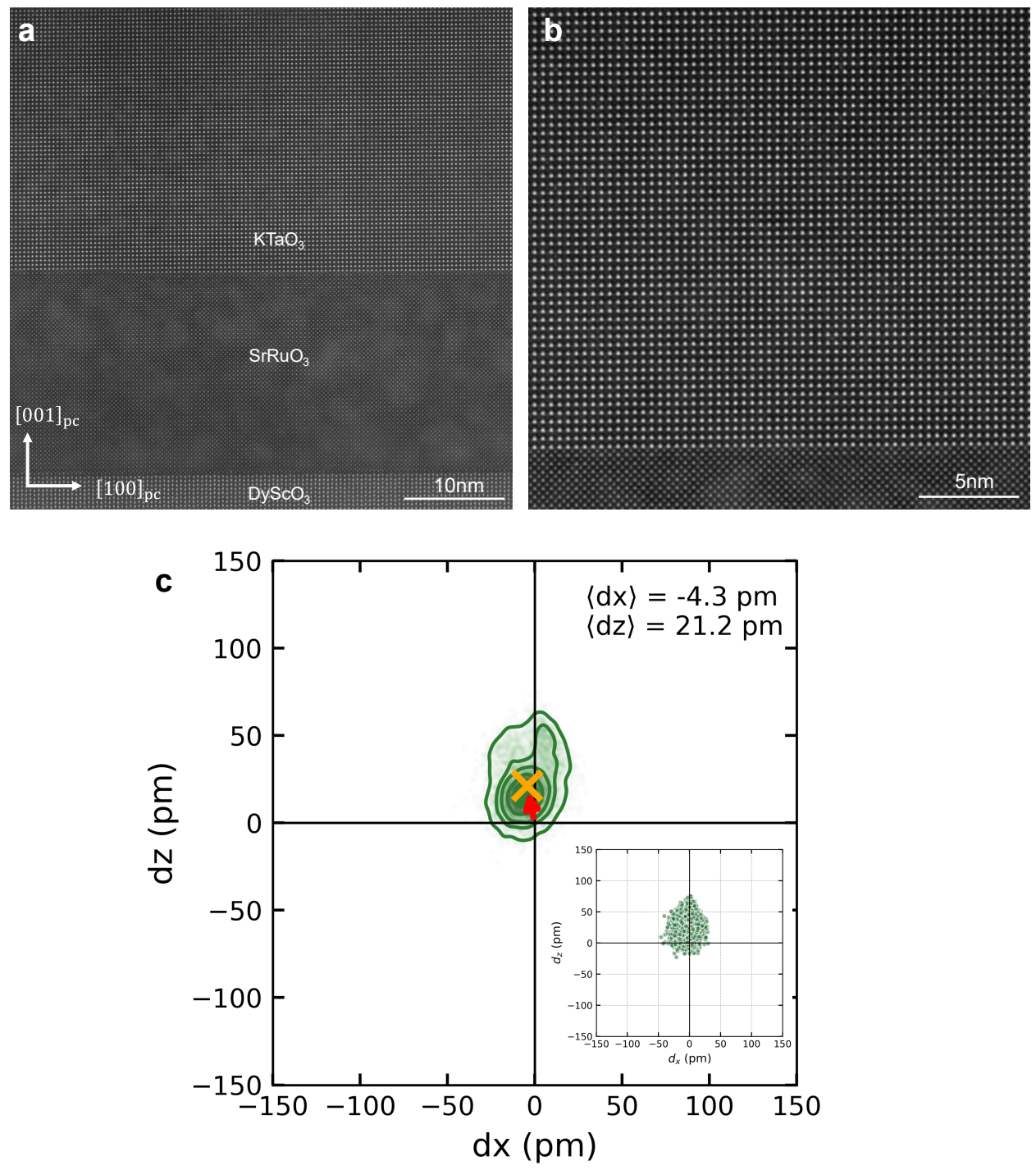

Fig. S10. Statistical analysis of atomic displacements in the $DyScO_3$-based capacitor heterostructure measured to exhibit ferroelectric switching. **a,b** HAADF-STEM images of the $KTaO_3/SrRuO_3/DyScO_3$ heterostructure from the same device for which switching is shown in Fig. S14. **c** Pooled two-dimensional distribution of atomic displacements in the analyzed region of the switched device. The contour lines summarize the distribution, and the orange marker denotes the mean displacement.

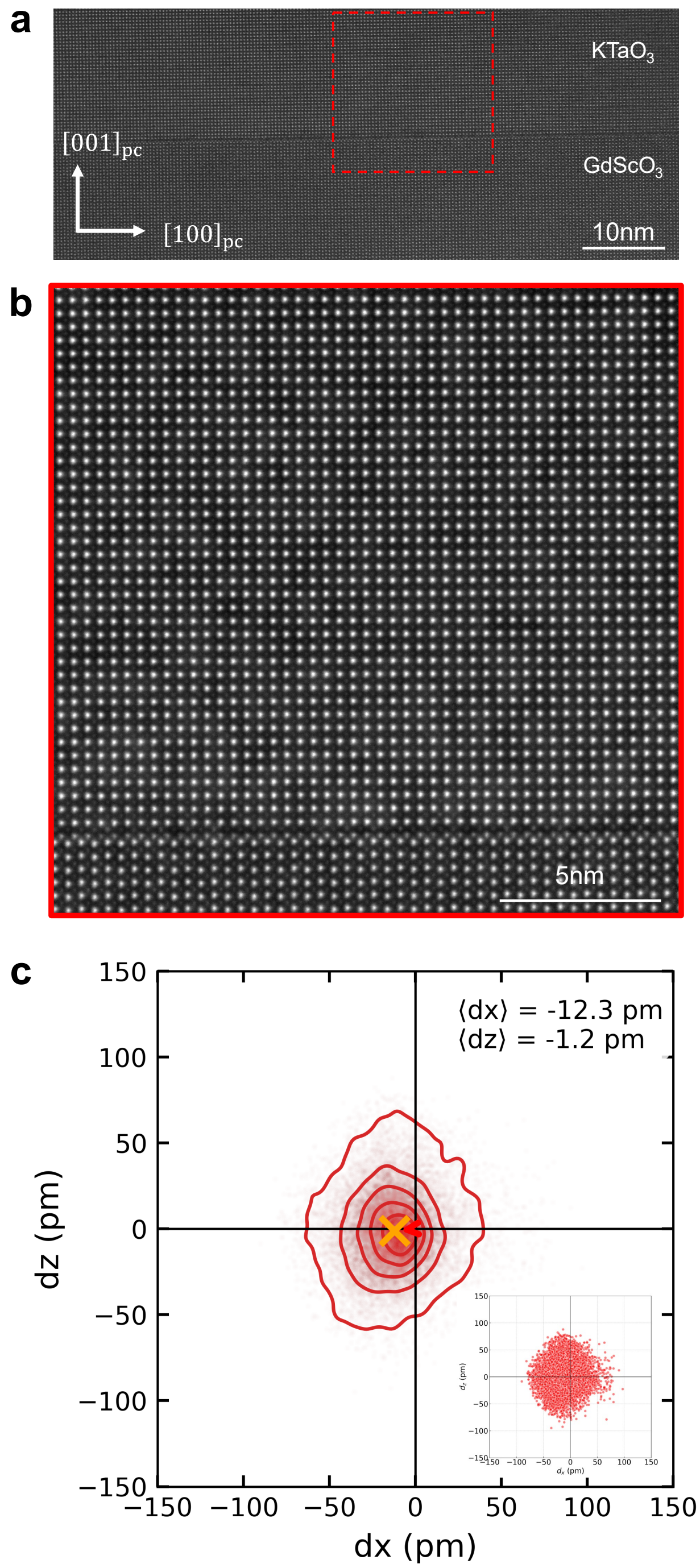

Fig. S11. Statistical analysis of atomic displacements in $KTaO_3$ grown on $GdScO_3$. **a,b** HAADF-STEM images of the $KTaO_3/GdScO_3$ heterostructure. **c** Pooled two-dimensional distribution of K-column displacements in the analyzed regions. The contour lines summarize the distribution, and the orange marker denotes the mean displacement.

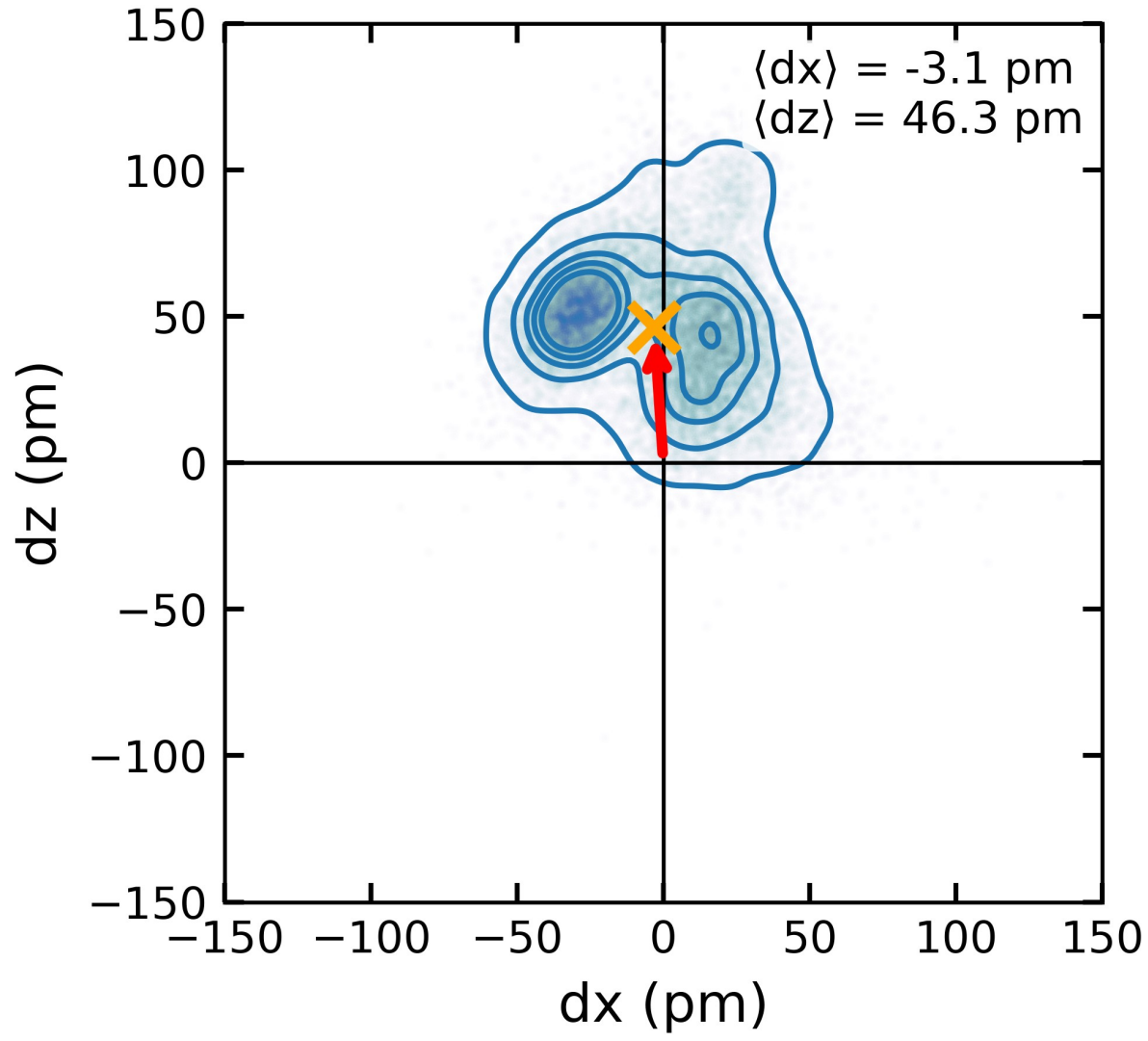

Fig. S12. Statistical analysis of atomic displacements in $KTaO_3$ grown on $SrTiO_3$. Pooled two-dimensional displacement distribution compiled from multiple HAADF-STEM images acquired from several analyzed regions. The contour plot shows two dominant populations with opposite in-plane displacements and similar positive out-of-plane components. The orange marker denotes the mean displacement.

VI. BORN EFFECTIVE CHARGES FROM FIRST PRINCIPLES

Born effective charges were calculated from the linear response within the VASP package. The Born effective charge Z^* matrices for each strain are shown in Table S5.

TABLE S5. Born effective charge Z^* matrices in $|e|$ for each element with different strain.

element	$KTaO_3$ (bulk)	$GdScO_3$ (-0.5 %)	$DyScO_3$ (-0.9 %)	$SrTiO_3$ (-2.1 %)
K	$\begin{pmatrix} 1.21 & 0.00 & 0.00 \\ 0.00 & 1.21 & -0.03 \\ 0.00 & 0.00 & 1.21 \end{pmatrix}$	$\begin{pmatrix} 1.27 & 0.00 & 0.00 \\ 0.00 & 1.19 & 0.00 \\ 0.00 & 0.04 & 1.19 \end{pmatrix}$	$\begin{pmatrix} 1.23 & 0.00 & 0.00 \\ 0.00 & 1.19 & 0.02 \\ 0.00 & 0.03 & 1.20 \end{pmatrix}$	$\begin{pmatrix} 1.25 & 0.00 & 0.00 \\ 0.00 & 1.22 & 0.01 \\ 0.00 & 0.01 & 1.21 \end{pmatrix}$
Ta	$\begin{pmatrix} 5.06 & 0.00 & 0.00 \\ 0.00 & 4.32 & 0.23 \\ 0.00 & -0.23 & 4.32 \end{pmatrix}$	$\begin{pmatrix} 4.99 & 0.00 & 0.00 \\ 0.00 & 8.22 & 0.04 \\ 0.00 & -0.13 & 8.03 \end{pmatrix}$	$\begin{pmatrix} 4.85 & 0.00 & 0.00 \\ 0.00 & 7.99 & 0.03 \\ 0.00 & -0.14 & 7.44 \end{pmatrix}$	$\begin{pmatrix} 4.65 & 0.00 & 0.00 \\ 0.00 & 7.66 & 0.11 \\ 0.00 & -0.06 & 6.59 \end{pmatrix}$
O	$\begin{pmatrix} -1.25 & 0.00 & 0.00 \\ 0.00 & -3.51 & -0.07 \\ 0.00 & -0.16 & -0.99 \end{pmatrix}$	$\begin{pmatrix} -1.21 & 0.00 & 0.00 \\ 0.00 & -6.40 & -0.11 \\ 0.00 & -0.31 & -1.52 \end{pmatrix}$	$\begin{pmatrix} -1.09 & 0.00 & 0.00 \\ 0.00 & -6.10 & -0.05 \\ 0.00 & -0.15 & -1.50 \end{pmatrix}$	$\begin{pmatrix} -1.06 & 0.00 & 0.00 \\ 0.00 & -5.93 & -0.03 \\ 0.00 & -0.06 & -1.40 \end{pmatrix}$
O	$\begin{pmatrix} -3.77 & 0.00 & 0.00 \\ 0.00 & -1.03 & -0.30 \\ 0.00 & 0.30 & -1.03 \end{pmatrix}$	$\begin{pmatrix} -3.83 & 0.00 & 0.00 \\ 0.00 & -1.46 & -0.01 \\ 0.00 & 0.15 & -1.59 \end{pmatrix}$	$\begin{pmatrix} -3.82 & 0.00 & 0.00 \\ 0.00 & -1.60 & -0.02 \\ 0.00 & 0.09 & -1.52 \end{pmatrix}$	$\begin{pmatrix} -3.70 & 0.00 & 0.00 \\ 0.00 & -1.53 & -0.01 \\ 0.00 & 0.07 & -1.41 \end{pmatrix}$
O	$\begin{pmatrix} -1.25 & 0.00 & 0.00 \\ 0.00 & -0.99 & 0.16 \\ 0.00 & 0.07 & -3.51 \end{pmatrix}$	$\begin{pmatrix} -1.22 & 0.00 & 0.00 \\ 0.00 & -1.55 & 0.16 \\ 0.00 & 0.25 & -6.11 \end{pmatrix}$	$\begin{pmatrix} -1.18 & 0.00 & 0.00 \\ 0.00 & -1.48 & 0.02 \\ 0.00 & 0.18 & -5.62 \end{pmatrix}$	$\begin{pmatrix} -1.14 & 0.00 & 0.00 \\ 0.00 & -1.42 & -0.08 \\ 0.00 & 0.03 & -4.98 \end{pmatrix}$

VII. DERIVATION OF OPTICAL SECOND HARMONIC GENERATION (SHG) POLARIMETRY EXPRESSION FOR THE TETRAGONAL PHASE *4mm*

Symmetry analysis of the $KTaO_3$ thin films was performed through fitting SHG polarimetry to analytical model as described below. The orientation of the substrate with respect to lab coordinates (x_1, x_2, x_3) are described in Fig. 4a. All measurements are done in reflection geometry.

The induced nonlinear polarization, $P^{2\omega}$,and the incident electric field, E^{ω}, is related in a second harmonic generation process as,

$$P_i^{2\omega} \propto d_{ijk} E_j^{\omega} E_k^{\omega} \tag{22}$$

where, the proportionality constants depend on the incident beam fluence, beam shape, and Fresnel coefficients at the film-air, film-substrate interfaces and thickness of the films[12]. d_{ijk} is the nonlinear susceptibility tensor and depends on the symmetry of the material in the crystal physics. For tetragonal $4mm$ point group symmetry, d_{ijk} has the following form in Voigt notation:

$$d_{ij} = \begin{pmatrix} 0 & 0 & 0 & 0 & d_{15} & 0 \\ 0 & 0 & 0 & d_{15} & 0 & 0 \\ d_{31} & d_{31} & d_{33} & 0 & 0 & 0 \end{pmatrix} \tag{23}$$

The linearly polarized incident electric field E^{ω} is rotated by φ and hence has the form $E_{\omega} = (E_{\omega}\cos(\psi), E_{\omega}\sin(\psi), 0)$. Taking into account the oblique incidence measurement geometry, the electric field in the crystal axes coordinates can be written as $(E_0\cos(\varphi)\cos(\theta), E_0\sin(\varphi), -E_0\cos(\varphi)\sin(\theta))$, where θ is the angle of incidence.

The induced polarization in the crystal physics coordinates, $i = (1, 2, 3)$, can be calculated through equation 3, which can be rotated back to (x_1, x_2, x_3) coordinates to give the p and s-polarized components of SHG ($p||X$ and $s||Y$). The p and s-polarized SHG intensities can be expressed as follows:

$$\begin{gathered} I_p^{2\omega} \propto \left(P_p^{2\omega}\right)^2 \propto \left((2d_{15} - d_{31} - d_{33})\cos[\varphi]^2 - 2d_{31}\sin[\varphi]^2\right)^2 \\ I_s^{2\omega} \propto \left(P_s^{2\omega}\right)^2 \propto d_{15}^2 \sin[2\varphi]^2 \end{gathered} \tag{24}$$

VIII. INCIDENT POWER DEPENDENCE SHG MEASUREMENTS

Figure S11 shows the SHG intensity as a function of incident laser power measured for strained and relaxed $KTaO_3$ films on $SrTiO_3$, $DyScO_3$, and $GdScO_3$ substrates. While strained films on $SrTiO_3$ and $DyScO_3$ substrates show SHG beyond bare substrate, the relaxed films and the films grown on $GdScO_3$ do not show SHG signal beyond their respective bare substrates.

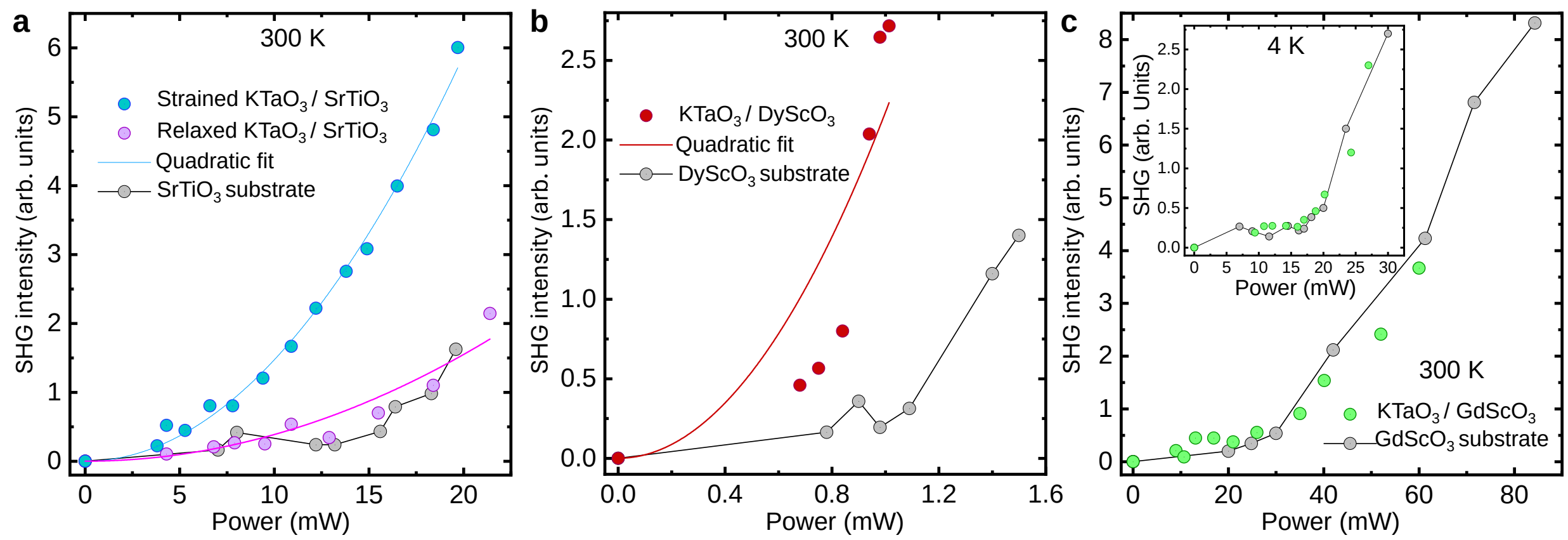

Fig. S13. **a**. SHG intensity as a function of incident laser power measured for strained and relaxed $KTaO_3$ films on $SrTiO_3$ substrate compared to similar measurement on a bare $SrTiO_3$ substrate. Thinner strained $KTaO_3$ film (9 nm thick) shows a prominent SHG response quadratically scaling with the incident fundamental laser power. Thicker relaxes $KTaO_3$ film (19 nm thick), however, shows no SHG response relative to the bare $SrTiO_3$ substrate. **b** Quadratically scaling power depedent SHG response of strained $KTaO_3$ films grown on $DyScO_3$ substrate. **c** Power dependent SHG measured on strained $KTaO_3$ film grown on $GdScO_3$ shows no SHG response different from measurements on bare $GdScO_3$ substrate.

IX. PIEZO FORCE MICROSCOPY OF $KTaO_3$ ON $DyScO_3$

Figure SS14 shows the $KTaO_3$/$SrRuO_3$/$DyScO_3$ capacitor hetero-structure is investigated using PFM. A 180° phase reversal for the out-of-plane PFM response is observed. The structural characterization details of this sample are discussed in Supplemental Discussion III.

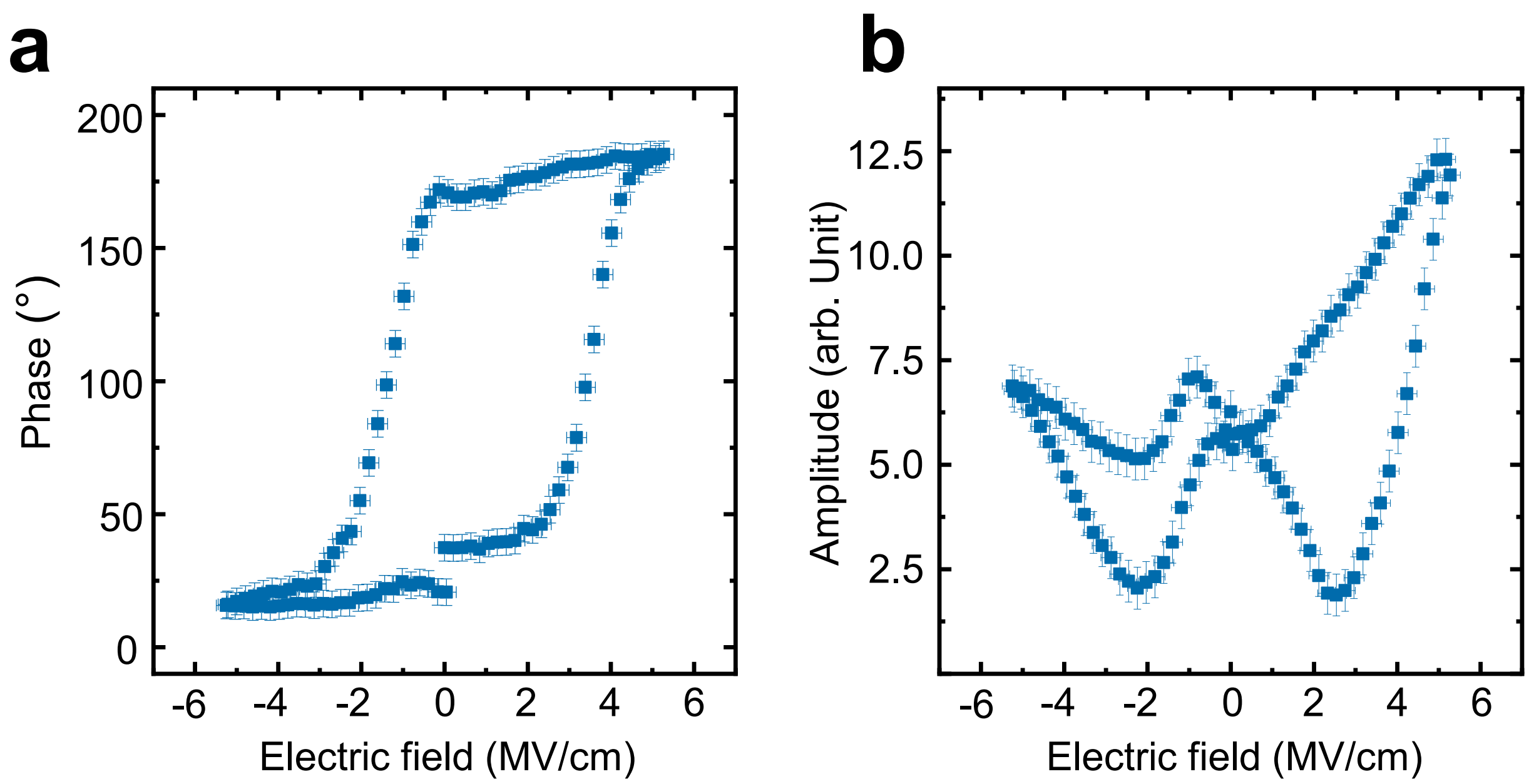

Fig. S14. **a** PFM switching using the $KTaO_3/SrRuO_3/DyScO_3$ sample showing a upward build-in field. **b** Magnitude of piezo response.

REFERENCES

[1] J. P. Perdew, A. Ruzsinszky, G. I. Csonka, O. A. Vydrov, G. E. Scuseria, L. A. Constantin, X. Zhou, and K. Burke, "Restoring the Density-Gradient Expansion for Exchange in Solids and Surfaces," Phys. Rev. Lett. **100**, 136406 (2008).

[2] G. Kresse and J. Furthmüller, "Efficient iterative schemes for ab initio total-energy calculations using a plane-wave basis set," Phys. Rev. B **54**, 11169–11186 (1996).

[3] G. Kresse and D. Joubert, "From ultrasoft pseudopotentials to the projector augmented-wave method," Phys. Rev. B **59**, 1758–1775 (1999).

[4] A. Togo, L. Chaput, T. Tadano, and I. Tanaka, "Implementation strategies in phonopy and phono3py," J. Phys. Condens. Matter **35**, 353001 (2023).

[5] M. J. Haun, E. Furman, S. Jang, H. McKinstry, and L. Cross, "Thermodynamic theory of $PbTiO_3$," Journal of Applied Physics **62**, 3331–3338 (1987).

[6] N. Pertsev, A. Zembilgotov, and A. Tagantsev, "Effect of mechanical boundary conditions on phase diagrams of epitaxial ferroelectric thin films," Physical review letters **80**, 1988 (1998).

[7] H. Uwe and T. Sakudo, “Electrostriction and stress-induced ferroelectricity in $KTaO_3$,” Journal of the Physical Society of Japan **38**, 183–189 (1975).

[8] D. S. Tanner, P.-E. Janolin, and E. Bousquet, “Strain-engineered divergent electrostriction in $KTaO_3$,” Physical Review B **106**, L060102 (2022).

[9] J. W. Fleming, M. J. Weber, G. W. Day, A. Feldman, B. H. Chai, M. G. Kuzyk, W. R. Holland, C. F. Rapp, M. Minden, D. T. Moore, *et al.*, *Handbook of optical materials* (CRC press, 2018).

[10] H. Bouafia, S. Hiadsi, B. Abidri, A. Akriche, L. Ghalouci, and B. Sahli, “Structural, elastic, electronic and thermodynamic properties of $KTaO_3$ and $NaTaO_3$: Ab initio investigations,” Computational materials science **75**, 1–8 (2013).

[11] S. Cabuk, “Ab initio volume-dependent elastic and lattice dynamics properties of $KTaO_3$,” physica status solidi (b) **247**, 93–97 (2010).

[12] R. Zu, B. Wang, J. He, J.-J. Wang, L. Weber, L.-Q. Chen, and V. Gopalan, “Analytical and numerical modeling of optical second harmonic generation in anisotropic crystals using shaarp package,” npj Computational Materials **8**, 246 (2022).
